# Of sequences and images - diversity and quantity of Arctic epipelagic zooplankton by an integrative approach

**DOI:** 10.1093/plankt/fbaf059

**Published:** 2025-11-28

**Authors:** Silke Laakmann, Astrid Cornils, Katja Metfies, Julian Koplin, Stefan Neuhaus, Carina Bunse, Barbara Niehoff, Hauke Flores

**Affiliations:** Plankton Ecology, Helmholtz Institute for Functional Marine Biodiversity at the University of Oldenburg (HIFMB), Im Technologiepark 5, 26129 Oldenburg, Germany; Alfred Wegener Institute, Helmholtz-Centre for Polar and Marine Research (AWI), Am Handelshafen 12, 27570 Bremerhaven, Germany; Polar Biological Oceanography, Alfred Wegener Institute, Helmholtz-Centre for Polar and Marine Research (AWI), Am Handelshafen 12, 27570 Bremerhaven, Germany; Polar Biological Oceanography, Alfred Wegener Institute, Helmholtz-Centre for Polar and Marine Research (AWI), Am Handelshafen 12, 27570 Bremerhaven, Germany; Helmholtz Institute for Functional Marine Biodiversity at the University of Oldenburg (HIFMB), Im Technologiepark 5, 26129 Oldenburg, Germany; Polar Biological Oceanography, Alfred Wegener Institute, Helmholtz-Centre for Polar and Marine Research (AWI), Am Handelshafen 12, 27570 Bremerhaven, Germany; Research Institute tor Sustainability (RIFS) | at GFZ, Berliner Str. 130, 14467 Potsdam, Germany; Data Science Support, Alfred Wegener Institute, Helmholtz-Centre for Polar and Marine Research (AWI), Klüßmannstraße 3, 27570 Bremerhaven, Germany; Helmholtz Institute for Functional Marine Biodiversity at the University of Oldenburg (HIFMB), Im Technologiepark 5, 26129 Oldenburg, Germany; Department of Marine Sciences, University of Gothenburg, Medicinaregatan 7B, 413 90 Gothenburg, Sweden; Polar Biological Oceanography, Alfred Wegener Institute, Helmholtz-Centre for Polar and Marine Research (AWI), Am Handelshafen 12, 27570 Bremerhaven, Germany; Polar Biological Oceanography, Alfred Wegener Institute, Helmholtz-Centre for Polar and Marine Research (AWI), Am Handelshafen 12, 27570 Bremerhaven, Germany

**Keywords:** Zooplankton, ZooScan, Metabarcoding, Imaging, Integrative approach, Arctic Ocean, COI, 18S rRNA V4, 18S rRNA V9

## Abstract

Due to the high sensitivity of zooplankton to environmental fluctuations, monitoring their taxonomic composition, abundance and biomass is of high priority to identify changes in the ecosystem. Recent advances in imaging and molecular technologies promise to greatly accelerate the processing of samples to determine both the diversity and quantity of the zooplankton community. In our study, we analyzed the diversity and quantity of an epipelagic Arctic zooplankton community using multi-marker metabarcoding and imaging analysis (ZooScan). We identified a total of 11 phyla and 58 species in the northern Barents Sea and the Nansen Basin. Metabarcoding identified more taxa than image analysis, while imaging provided quantitative information on abundance and biomass. Multivariate analyses revealed overall the same significant environmental drivers (temperature and percentage of Polar Surface Water in the sampling depth layer) explaining the similarity and spatial distribution of the zooplankton community. For all approaches, similar spatial patterns of the zooplankton community were found. Abundance, biovolume and biomass decreased with increasing latitude within the analyzed regions. Based on this study, we recommend ZooScan image analysis in combination with COI metabarcoding for future monitoring of Arctic zooplankton diversity and quantification to ensure the detection of changes in both aspects of these communities.

## INTRODUCTION

Zooplankton play a significant role in maintaining pelagic ecosystem functioning. They are drivers in nutrient cycling and carbon sequestration, and they serve as primary food source for many marine organisms, such as various invertebrates, fish and birds (Darnis and Fortier, 2012; Steinberg and Landry, 2017; Balazy *et al.*, 2023). Understanding the diversity, community structure and abundance of marine zooplankton is important, as it reflects the complex (ecological) interactions that structure marine ecosystems. Species composition and abundance respond to environmental changes, climate and human activities (Berry *et al.*, 2019, 2023; Ndah *et al.*, 2022). The high sensitivity to temperature, sea-ice coverage and salinity makes zooplankton important indicators of environmental change (Hays *et al.*, 2005; Dalpadado *et al.*, 2012; Ershova *et al.*, 2015, 2021a). As a consequence, monitoring zooplankton serves as an early warning system for environmental variations and disturbances, helping scientists and policymakers to anticipate and address potential shifts in marine ecosystems (Snoeijs-Leijonmalm *et al.*, 2020; ICES, 2022).

Monitoring is a high priority in areas that are highly vulnerable and impacted by environmental change, particularly in pristine ecosystems (Ratnarajah *et al.*, 2023). Examples are the Arctic Ocean and adjacent regions that are currently undergoing a rapid transformation. The two major processes toward a new Arctic are sea-ice decline and the so-called “Atlantification” of the Eurasian basin, where progressing Atlantic influence plays a much greater role than before (Polyakov *et al.*, 2017). Changes in the geographical and vertical distribution and behavior of the zooplankton community have been predicted (Dalpadado *et al.*, 2012; Eriksen *et al.*, 2017; Flores *et al.*, 2023). However, our knowledge of interannual variations in zooplankton dynamics in the context of the seasonal cycle in the Arctic Ocean and its adjacent areas is still sparse although being essential for understanding and quantifying the impact of climate change.

Traditionally, zooplankton monitoring has been conducted using plankton net samples and microscopic identification based on morphological features, which is time consuming, labor intensive and sensitive to human biases (Mackas and Beaugrand, 2010; Boersma *et al.*, 2015). Compared to microscopy, imaging technologies and molecular approaches allow for a faster taxonomic processing of net samples and readily available data in digital format (Gorsky *et al.*, 2010; Bucklin *et al.*, 2021; Cornils *et al.*, 2022; Pierella Karlusich *et al.*, 2022; Ohnesorge *et al.*, 2023, 2024).

Imaging has become an essential tool in plankton studies over the last two decades and is ideal for monitoring purposes. A wide range of imaging systems are now available (for review see Lombard *et al.*, 2019). For processing of zooplankton net samples, two systems with high-resolution images are available, the ZooScan and the FlowCam Macro (Lombard *et al.*, 2019). A major advantage of these systems compared to microscopy is that size measures in each object are generated automatically (Gorsky *et al.*, 2010), which allows for an estimate of individual biovolume and, subsequently, biomass applying conversion factors (Ikeda and Skjoldal, 1989; Kiørboe, 2013). However, automatic species identification in both systems is still limited (Romagnan *et al.*, 2016; Ershova *et al.*, 2024) mainly due to the two-dimensional image and damaged organisms. In the present study, we have chosen the ZooScan, a laboratory-based scanning system that allows scanning aliquots of preserved net samples (Gorsky *et al.*, 2010) and extracting single-object images. The objects on the images can then be classified semi-automatically with the web application EcoTaxa (Picheral *et al.*, 2017), combining automated prediction by a supervised deep learning algorithm with manual validation and correction to gain the best taxonomic resolution possible. This yields similar results in taxonomic composition, abundance and biomass in the Atlantic Arctic Ocean with imaging as with microscopy (Cornils *et al.*, 2022). Here, we build on these results and combine the imaging with multi-marker metabarcoding.

Metabarcoding involves using high-throughput sequencing of short-read fragments of orthologous genes (barcodes) to identify the taxa present in a sample to a certain taxonomic level. One of the advantages of metabarcoding is the ability to distinguish among species across diverse taxa. This includes morphologically challenging groups such as young developmental stages, fragile organisms (such as gelatinous taxa shrink and deform in fixation solution) and rare, non-indigenous and cryptic species in bulk zooplankton samples, resulting in higher species numbers, and thus diversity, compared to morphological investigations (Lindeque *et al.*, 2013; Hirai *et al.*, 2015; Mohrbeck *et al.*, 2015; Abad *et al.*, 2016; Stefanni *et al.*, 2018; Bucklin *et al.*, 2019; Ershova *et al.*, 2023). Today, multi-marker metabarcoding approaches, combining species-specific markers such as mitochondrial cytochrome *c* oxidase subunit I (COI) and markers resolving on higher taxonomic level such as nuclear ribosomal 18S or 28S rRNA subunits, are frequently applied and recommended to assess metazoan communities in marine ecosystems (Clarke *et al.*, 2017; Günther *et al.*, 2018; Stefanni *et al.*, 2018; Ershova *et al.*, 2021b, 2023; Ohnesorge *et al.*, 2023, 2024). However, any molecular genetic approach can only resolve the taxonomic levels to the extent of its reference database for the markers identifying the taxa in the ecosystem to be studied. Furthermore, metabarcoding does not provide comprehensive information on population structure (i.e. size and developmental stages) as well as on biomass and abundance (reviewed by Laakmann *et al.*, 2020). Recently, relationships between organism biomass and sequence numbers from metabarcoding approaches have been established (Hirai *et al.*, 2017; Krehenwinkel *et al.*, 2017; Ershova *et al.*, 2021b, 2023; Weiß *et al.*, 2024). These relationships have proven to be region, taxa and protocol dependent, and thus, it remains challenging to convert sequence numbers to meaningful biomass estimates. We propose that the integration of imaging and metabarcoding circumvents the limitations of each method, and obtains high taxonomic resolution and, at the same time, taxa-specific abundance, biovolume and size structure. Considering the effort required for microscopic analyses alone, a joint application could also speed up the processing of samples. Application to an aliquot (split) of the same sample also enables cross-validation of the two methodologies.

This study focuses on the identification of epipelagic Arctic zooplankton communities in the Central Arctic Ocean. We examine patterns in species diversity, community structure and their environmental drivers by applying metabarcoding of COI, 18S rRNA variable regions 4 (V4) and 9 (V9), together with the ZooScan imaging.

## MATERIAL AND METHODS

### Sampling

**Fig. 1:**
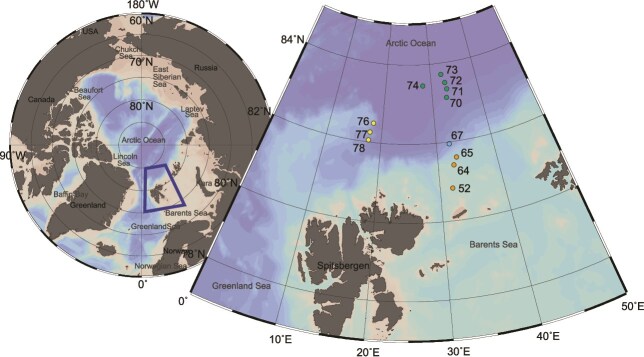
Stations, zooplankton samples have been collected in the upper 100 m by Rectangular Midwater Trawl 1 (RMT1; 1-m^2^ aperture, 320 μm) in June and July 2017 in the Nansen Basin during PS106.2. Barents Sea (BS) stations: 52, 64, 65; Southern Nansen Basin (SNB) station: 67; Central Nansen Basin (NB) stations 70, 71, 72, 73, 74; Yermak Plateau (YP) stations: 76, 77, 78.

During the expedition PS106.2 with RV *Polarstern* from 23 June to 20 July 2017, zooplankton samples were taken on a transect from the shelf and slope of the Barents Sea (BS: stations 52, 64 and 65, in orange) into the southern (SNB: station 67, in blue) and central Nansen Basin (NB: stations 70, 71, 72, 73 and 74 in green), and close to the Yermak Plateau (YP: stations 76, 77 and 78, in yellow; Table I, Fig. 1). Double oblique hauls were performed with a rectangular midwater trawl (RMT) over a depth range of 100 m to the surface. The RMT consists of a macrozooplankton net with a nominal net opening of 8 m^2^ and a mesh size of 5 mm (RMT8), and a mesozooplankton net with a nominal mouth opening of 1 m^2^ and a mesh size of 320 μm (RMT1). The mean towing speed ranged from 2 to 3 knots (Macke and Flores, 2018). The volume of filtered water was estimated after Roe and Shale (1979) based on the effective net opening and the speed of the ship and ranged from 651 to 1443 m^3^ (see dataset Cornils *et al.*, 2024). In the present study, we analyzed the samples collected with the RMT1 only. Onboard, the samples were split in two halves with a Motoda plankton splitter (Motoda, 1959). One half was preserved in 4% buffered formaldehyde–seawater solution for quantitative image analysis. The second half was fractionated in four size classes using sieves (0.5–1–2–4 mm) and frozen in petri dishes at −20°C for dry mass measurements and metabarcoding (S1). For the comparison of the metabarcoding and imaging results, size fractions <0.5 mm were not analyzed, as they were influenced by debris rinsed down from larger size classes and marine snow, which affect both taxonomic results of metabarcoding and dry mass estimates. Prior to the metabarcoding analysis, each of the four size fractions was freeze dried for 48 h and dry mass (DM) was estimated to the nearest microgram. Subsequently, the samples were ground and homogenized with piston and mortar.

**Table I:** Rectangular Midwater Trawl 1 (RMT1) stations during the expedition PS106.2

**Station**	**Date**	**Time (UTC)**	**Latitude**	**Longitude**	**Bottom depth (m)**
52	29.06.2017	14:41	80.82638	31.953966	135
64	01.07.2017	14:48	81.41416	32.612201	204.4
65	02.07.2017	04:43	81.59516	33.207016	553
67	03.07.2017	12:18	81.95435	32.330701	2818.3
70	05.07.2017	20:58	83.11927	32.924238	3813.4
71	06.07.2017	05:32	83.33400	33.237782	3902.6
72	06.07.2017	12:39	83.50125	32.981169	3982.7
73	07.07.2017	10:38	83.71395	32.337495	4022.3
74	08.07.2017	12:26	83.46790	28.085239	4049.1
76	10.07.2017	08:25	82.48965	18.224139	2277.8
77	10.07.2017	17:15	82.2445	17.782107	2024.9
78	11.07.2017	03:32	82.05043	17.643661	1849.4

### Image analyses

In the laboratory, the subsamples preserved for quantitative analyses were size fractionated using a sequence of sieves (0.5–1–2–4 mm, S1), primarily to avoid overlapping of large and small organisms on the scanning surface, which can lead to biases in abundance of small organisms. Additionally, the specific size classes were initially chosen to match those for parallel dry mass and metabarcoding analyses. Although the molecular data were later pooled, the size separation is standard practice in ZooScan workflows (Gorsky *et al.*, 2010; Cornils *et al.*, 2022). Each size fraction was then split into aliquots using a Motoda splitter to a minimum of 1/256, resulting in *ca*. 1000 particles per subsample as recommended by Gorsky *et al.* (2010). The final aliquots were digitized using the ZooScan, a waterproof flatbed scanner (Model Biotom, Hydroptic, France; 2400-dpi resolution). The scanned images were then processed using ZooProcess, a macro written in ImageJ macro language (Schneider *et al.*, 2012). ZooProcess links the scans with associated metadata and cuts the full scan into single-object images. Images that contained multiple or overlapping organisms were cut manually in the software and were re-processed.

**Fig. 2:**
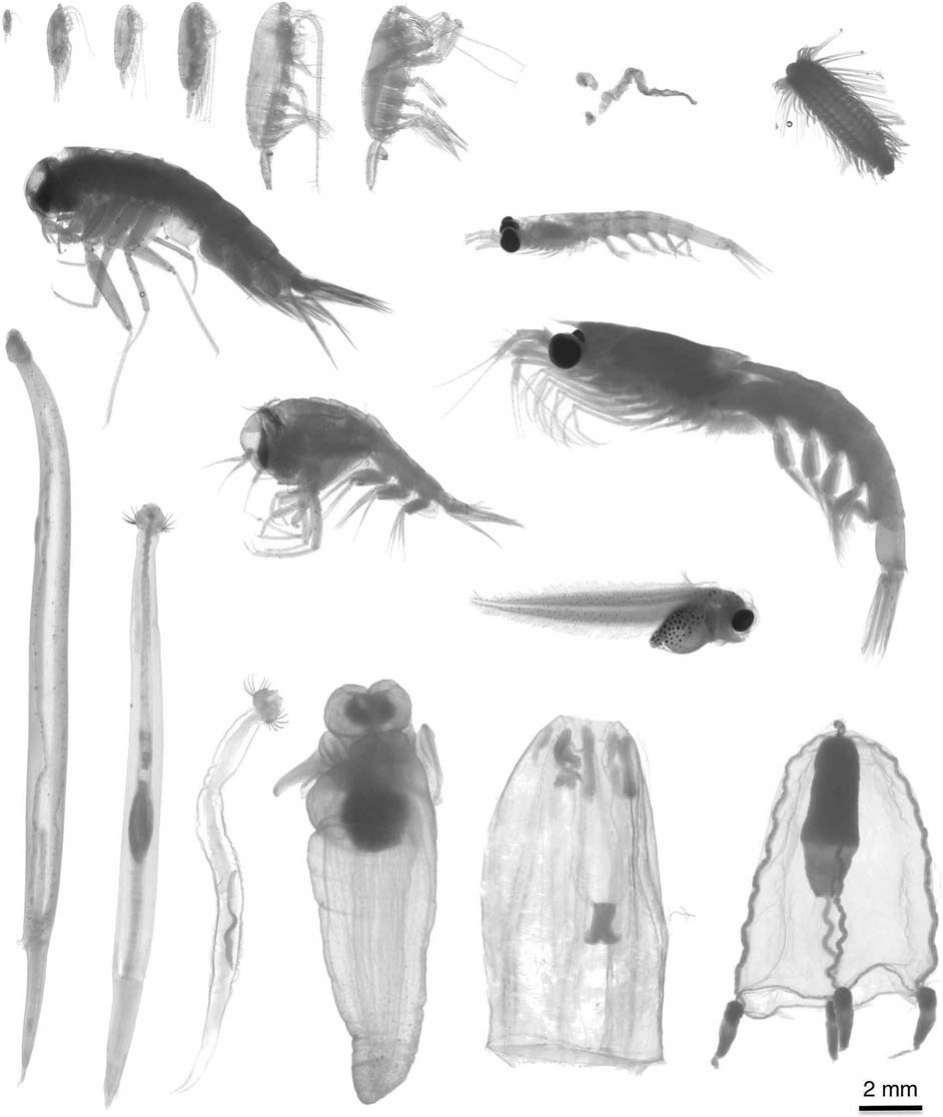
Selected taxa from the ZooScan analyses. Top row: *Pseudocalanus* sp., *Metridia longa*, *Calanus finmarchicus*, *Calanus glacialis*, *Calanus hyperboreus*, *Paraeuchaeta* sp., *Oikopleura* sp. (trunk, tail), Polychaeta; 2nd row: *Themisto libellula*, *Thysanoessa longicaudata*; 3rd row: *Themisto abyssorum*, *Liparis* sp., *Thysanoessa inermis*; bottom row: *Parasagitta elegans*, *Eukrohnia hamata*, *Pseudosagitta maxima*, *Clione limacina*, *Aglantha digitale*, *Euphysa* sp.

After the upload of all images to the web application EcoTaxa (Picheral *et al.*, 2017), a built-in deep learning model trained for ZooScan images was applied to automatically predict taxonomic categories using a training set of validated Arctic zooplankton images (Cornils *et al.*, 2022). The predictions were then confirmed manually or, if necessary, corrected by taxonomic experts. In total, 14 371 single-object images were uploaded onto EcoTaxa. On the images, 7559 presented objects were not assigned to complete zooplankton organisms (e.g. artifact, detritus, bubbles, body parts) and were discarded. All organisms were classified to the lowest possible taxonomic level (Fig. 2). Due to the orientation of the objects on the images, however, not all organisms could be identified to species level (see S2, S3). Data from the three *Calanus* species were assigned according to their prosome length as described in Cornils *et al.* (2022).

During the processing of the images, size measures for each object are generated automatically (area, maximum length and width; Gorsky *et al.*, 2010, Vandromme *et al.*, 2012), which allows for an estimate of individual biovolume, and, subsequently, biomass applying conversion factors (e.g. Ikeda and Skjoldal, 1989; Kiørboe, 2013; Cornils *et al.*, 2022). Biovolume (BV; mm^3^) of each organism was calculated with the following formula: BV = 4/3^*^π^*^(major/2^*^(minor/2)^2^). In the formula, “major” is the primary and “minor” the secondary axis of an ellipse that describes the body shape of most planktonic organisms, in particular copepods. Individual dry mass (mg DM) was estimated by converting the biovolume of each individual into dry mass using conversion factor for Arctic zooplankton (Cornils *et al.*, 2022 and references therein), assuming that zooplankton organisms are neutrally buoyant with a specific density of 1 g cm^3^ (Postel *et al.*, 2000 and references therein), and thus, the biovolume equals the wet mass. The conversion factor for Arctic copepods (0.16) overestimates the biomass of *Calanus hyperboreus* 2-fold (figure 6 in Cornils *et al.*, 2022). Consequently, we reduced the conversion factor from wet weight to dry weight for *C. hyperboreus* to 0.08. Abundances (number of individuals/m^3^), biovolume (mm^3^/m^3^) and biomass (mg DM/m^3^) were calculated for the from the sum of image counts, individual biovolumes or dry masses per taxon and station divided by the volume of filtered water.

### Molecular genetic analyses

Prior to DNA extraction, each freeze-dried size fraction was homogenized manually by stirring and shaking the ground samples. Then, 25 mg of the homogenized sample was placed in a 1.5-mL microcentrifuge tube and the genomic DNA was extracted with the QIAGEN DNeasy blood and tissue kit (Qiagen GmbH, Hilden, Germany) following the manufacturer’s protocol for tissue samples (S1). For this, 180 μL ATL buffer and 20 μL Proteinase K were added to the 1.5-mL microcentrifuge tubes containing 25 mg of the grinded samples and incubated at 56°C with repeated vortexing steps. Finally, elution was done using a 200-μL AE buffer with 1-min incubation time at room temperature and centrifugation for 1 min at 8000 rpm. DNA concentration of the extracts was analyzed using a fluorometer (QuantiFluorR dsDNA System; Promega, USA) including the measurement of a blank after all two measurements.

The two nuclear gene fragments 18S rRNA V4 and V9 and one mitochondrial gene fragment, cytochrome *c* oxidase subunit I (COI), were prepared for sequencing following a modified protocol of the 16S rRNA Metagenomic Sequencing Library Preparation Guide from Illumina (Illumina, 2013). The first polymerase chain reaction (PCR) was conducted in triplicates for all the samples with Illumina overhang primers. In all PCRs, positive and negative samples were included. The detailed protocols for library preparation, sequencing, sequence processing and sequence data analysis including taxa assignment are described in detail in the supplementary data (S11).

The sequencing depth varied between the size fraction. For the subsequent analysis, we applied a scaling procedure to obtain normalized sequence read abundances (NSRAs) of the amplicon sequence variants (ASVs) in order to combine all size fractions per station. First, the total number of reads per size fraction was scaled to the minimum sequencing depth of all fractions. Now, each fraction had the same total number of reads, but the original abundance ratios within each size fraction were retained. The scaled fractions were then merged for each station to obtain a summarized abundance matrix per station. This procedure avoids biases due to different sequencing depths and enables a fair consideration of all fractions without loss of taxonomic information due to rarefaction.

### Environmental parameters

Hydrographic measurements were obtained from CTD measurements conducted at zooplankton sampling stations (Heuzé *et al.*, 2018, S4). Stations 52, 64, 72 and 77 had no corresponding CTD station. Here, the profiles of the closest CTD stations 50 (80.517°N, 30.970°E), 65, 73 and 76, respectively, were chosen. For multivariate statistics, the values of temperature, salinity and fluorescence were averaged from the surface to 100-m depth (S4). From density and potential temperature values, water masses were identified for each depth according to Rudels *et al.* (2005) and Richter and Appen (2018). For the multivariate analysis, we calculated the relative contribution of Polar Surface Water (PSW) and Arctic Atlantic Water (AAW) over 100-m depth. Average sea-ice thickness was obtained from table 4 in Castellani *et al.* (2020).

### Data analysis

If not marked otherwise, all data analyses were conducted in the scientific R programming language in the Rstudio environment (R Core Team, 2025; RStudio Team, 2025) using primarily the R packages tidyverse (Wickham *et al.*, 2019) and vegan (Oksanen *et al.*, 2024). First, the NSRAs and the image-based abundance, biovolume and biomass were square-root transformed to account for skewness in count data.

Venn diagrams were generated to visualize the taxonomic overlap among the genetic markers and imaging abundance using the R package VennDiagram (Chen and Boutros, 2011). Taxa lists (phylum to species level) from each method were converted into presence–absence format, and shared as well as unique taxa were displayed as intersecting sets.

To compare the impact of genetic markers and quantitative image-based approaches in capturing alpha diversity and potential discrepancies in pattern across methods, the diversity indices were calculated. The pairwise Jaccard similarity and the Shannon diversity (H′) indices were calculated using the *vegdist()* and the *diversity()* function. Pearson correlation coefficients were computed between the resulting Jaccard matrices to assess consistency between markers and between Shannon diversity values across different markers to evaluate the consistency of diversity patterns of the stations. To determine the statistical significance of the correlations of the Jaccard and Shannon indices, *P*-values were computed using the *cor.test()* function, with *P*-values <0.05 being considered significant. The correlation structure, including both Jaccard and Shannon diversity indices, was visualized using a correlation heatmap generated by the *corrplot* package in R (Wei and Simko, 2017).

A principal component analysis (PCA) was performed using the *prcomp()* function to investigate the similarity of stations based on scaled environmental variables (see S4). To facilitate interpretation, Shannon diversity indices were incorporated as passive vectors in the PCA to examine the relationship between environmental variables and biodiversity.

The relationships between selected environmental variables and zooplankton quantitative measures (NSRA and image-based abundance, biovolume and biomass) were further explored using non-metric multidimensional scaling (nMDS). This analysis was based on Bray–Curtis similarity matrices, which are well suited to assess community composition based on abundance data. nMDS was performed using the *metaMDS()* function to explore community structure. Environmental vectors were added using the *envfit()* function, which included mean temperature, salinity and fluorescence, as well as mean sea-ice thickness, latitude and the thickness of the PSW layer.

To assess whether the predefined regional groups BS, SNB, NB and YP correspond to meaningful data structure in both PCA and nMDS analyses, we applied hierarchical clustering using Ward’s method and Euclidean distances (*hclust()*) with *k*-means clustering (*kmeans()*) to the first three PCA or nMDS axes. The number of clusters was evaluated based on visual inspection of dendrograms and within-cluster sum of squares.

## RESULTS

### Sequence assignments and taxonomic resolution

**Fig. 3:**
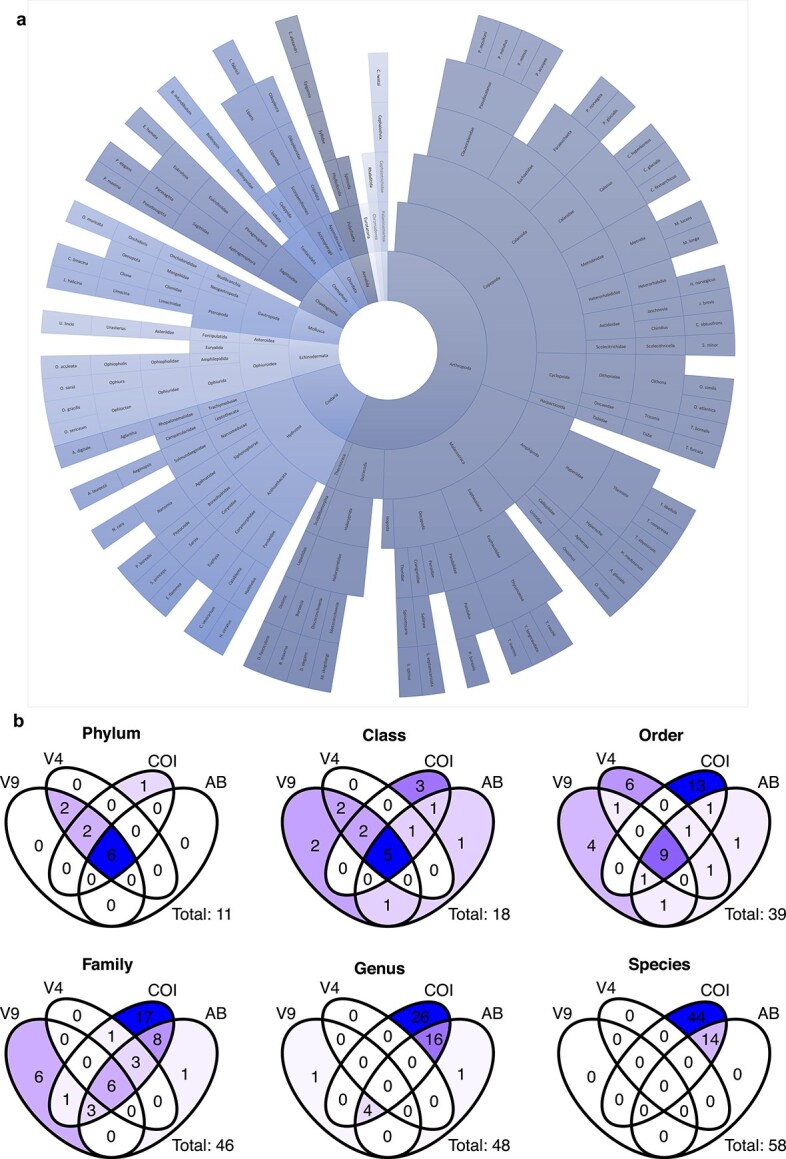
Overview of the number of Arctic zooplankton taxa identified across taxonomic levels. (a) Cumulative taxonomic resolution obtained from the integrated imaging–molecular approach, shown from phylum (innermost) to species (outermost level). (b) Comparison of taxa detected by individual approaches, from metabarcoding (18S rRNA V9, 18S rRNA V4, COI) and imaging based abundance (AB); illustrated as a Venn diagram.

For the sequence analysis, a total of 82.9% (V4), 91.5% (V9) and 54.7% (COI) of the sequence reads and 926 (V4), 498 (V9) and 11 870 (COI) ASVs were recovered for downstream analyses after the bioinformatics processing (Table II). We deleted 22 (V4), 2 (V9) and 57 (COI) ASVs as singletons and filtered the data set for metazoan phyla only. As a result, >91% of the reads and >28–74% of the ASVs were assigned to metazoans (Table II). For all three markers, reads could be assigned to the phylum (91.1–99.8%), class (91.0–99.2%), order (90.7–95.5%) and family (81.6–90.0%) level (Fig. 3b, S5). Only COI reads could be assigned in high percentages to the taxonomic ranks of genus (90.0%) and species (89.9%) level (S5). Sequencing depth for all three genetic markers was sufficient to recover metazoan ASVs and to identify zooplankton diversity in these samples, as demonstrated by ASV accumulation curves (S6).

**Table II:** Overview on raw, processed and assigned sequences and amplicon sequence variants (ASVs) for COI, 18S rRNA V4 and V9

**Marker**	**Raw reads**	**Processed reads**	**Total ASVs**	**After deletion of singletons**	**Metazoan reads**	**Metazoan ASVs**
COI	7 566 732	4 142 145 (54.7%)	11 870	4 142 088 reads; 11 813 ASVs	3 783 594 (91.3%)	3325 (28.1%)
18S rRNAV4	5 330 717	4 420 307 (82.9%)	926	4 420 286 reads; 904 ASVs	4 338 258 (98.1%)	452 (50.0%)
18S rRNA V9	8 319 478	7 614 262 (91.5%)	498	7 611 043 reads; 496 ASVs	7 577 053 (99.6)	367 (74.0%)

While similar percentages of reads were assigned for all three markers, the assignment of individual ASVs and their taxonomic resolution varied. A much higher percentage of 18S ASVs were assigned at least to the phylum or class level (49–74%) compared to COI, where only 27% were assigned (S5). However, COI outperformed 18S at the identification from order to species, detecting 58 species and 39 of the 43 families identified with molecular tools while V4 and V9 yielded only five families and none of the species (Fig. 3b, S7–9).

Overall, the three-marker molecular approach identified more taxa compared to the imaging (e.g. 11 vs. 6 phyla, and 58 vs. 14 species (Fig. 3b, S2, S3, S7–9)). A total of 6812 images could be identified as zooplankton organisms. However, not all organisms could be assigned to genus and species level as morphological diagnostic characters were not always visible (Fig. 2, Table III, S3). On the images, small species such as *Pseudocalanus* spp. and young developmental stages could not be identified to species level—here the metabarcoding approach complemented the image-based identifications. In total, only 1.6% of the ~5300 copepod images could not be assigned to the family level. For the Cnidaria, most images showed fragments of medusae and allowed only the identification of one species (*Aglantha digitale*; S3). All other Cnidaria were resolved to species level based on COI only. The Appendicularia were mostly broken due to net sampling. However, the images with appendicularian trunks confirmed the presence of the genus *Oikopleura.* With molecular tools, this genus was only detected with a very low read abundance using the V4 marker (S8).

**Table III:** Number of unique categories identified at each rank by image analysis with ZooScan and the genetic identification based on metabarcoding for the three marker genes COI, 18S rRNA V4 and 18S rRNA V4

**Taxonomic rank**	**ZooScan**	**COI**	**18S rRNA V4**	**18S rRNA V9**
Phylum	6	9	10	10
Class	9	13	12	10
Order	15	24	16	18
Family	21	39	16	10
Genus	21	46	4	-
Species	17	58	-	-

### Taxonomic composition and abundance

Imaging and multi-marker metabarcoding identified in total 58 species, 47 genera, 43 families, 31 orders and 16 classes from the 11 metazoan phyla Annelida, Arthropoda, Chaetognatha, Chordata, Cnidaria, Ctenophora (molecular only), Echinodermata (molecular only), Mollusca, Nemertea (COI only), Rotifera (18S rRNA only) and Nematoda (18S rRNA only) (Fig. 3, S2). Arthropoda was the most taxon-rich group, and of these, Copepoda was the most taxon-rich class, with seven Calanoida, two Cyclopoida and one Harpacticoida families (Fig. 3a, S2). Within the Calanoida, 15 species were identified, the most species rich being Clausocalanidae (*Pseudocalanus minutus*, *P. moultoni*, *Pseudocalanus acuspes* and *P. mimus*), followed by Calanidae (*Calanus finmarchicus*, *C. glacialis*, *C. hyperboreus*). The other five families were composed of two (Euchaetidae, Aetideidae) or one species (Metridinidae, Heterorhabdidae and Scolecitrichidae).

**Fig. 4:**
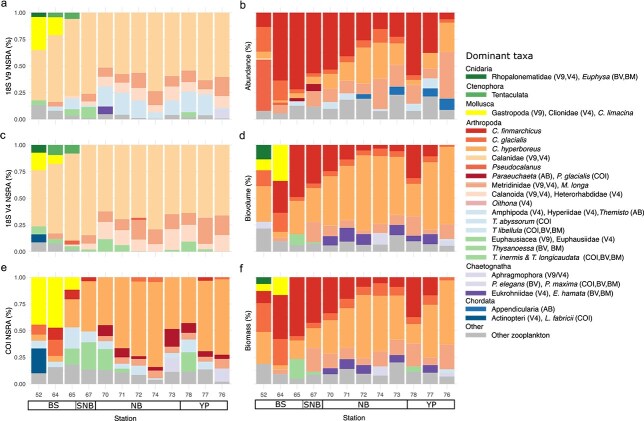
Taxonomic composition of the zooplankton, showing the five dominant taxa per station for each approach. (a) 18S rRNA V9 metabarcoding (V9), (b) image-based abundance (AB), (c) 18S rRNA V4 metabarcoding (V4), (d) image-based biovolume (BVD), (e) COI metabarcoding (COI), and (f) image-based biomass (BMF). NSRA: normalized sequence read abundance (%). The stations are ordered by region and increasing latitude in each region (BS: Barents Sea, SNB: southern Nansen Basin, NB: Nansen Basin, YP: Yermak Plateau).

**Fig. 5:**
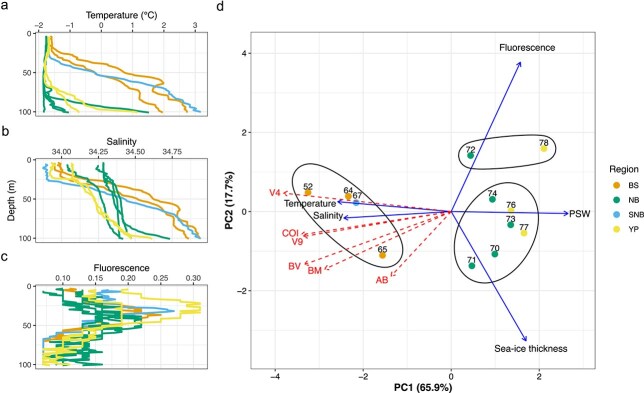
Environmental parameters in the 0–100-m depth layer during the study periods. (a) temperature, (b) salinity and (c) fluorescence profiles; data for (a), (b) and (c) were obtained from Heuzé *et al.* (2018). (d) PCA of environmental variables: temperature, salinity and fluorescence were averaged for this depth layer. Mean ice thickness was extracted from Castellani *et al.* (2020). Polar Surface Water (PSW) indicates the percentage share of PSW in this depth layer (see S4). Stations are grouped according to their distribution. BS: shelf and slope of Barents Sea, SNB: southern Nansen Basin, NB: Nansen Basin, YP: stations close to Yermak Plateau. The direction of the arrows indicates the relationship of the environmental parameters with the ordination. The 1 vectors for the Shannon diversity indices (red arrows) of the six different approaches (metabarcoding: 18S V9 and V4, COI; imaging: abundance (AB), biovolume (BV) and biomass (BM)) were added to the plot. Stations are grouped according to hierarchical clustering.

In all approaches, representatives of the family Calanidae were predominant, belonging to the five dominant taxa. With V4 and V9 they accounted for 50 to 72% of NSRA (Fig. 4a, c). With COI and image-based biovolume/biomass, *C. hyperboreus* accounted for 20 to 90% of all taxa at most stations (Fig. 4; except stations 52 and 64). Its dominance increased with latitude in the COI and imaging analyses. *Calanus glacialis* was the most common among the five dominant taxa at the BS stations (Fig. 4b, d–f). Only in the image-based analysis *C. finmarchicus* played a major role (10–75%, Fig. 4b), contributing on average 42% to the abundance. With COI, it contributed <10% to the NSRA. *Calanus finmarchicus* was particularly dominant at the BS slope (stations 64, 65), SNB and at southern NB (stations 70, 71) and YP (stations 78, 77). These stations were presumably less influenced by the PSW (Fig. 5d).

Apart from the Calanidae, *Metridia longa* (as Metridinidae in V4 and V9) was among the dominant taxa at most stations. *Metridia longa* contributed more to the community in SNB, NB and YP (9–41%) compared to BS (<10%). With the imaging approach, the calanoid copepod *Paraeuchaeta* spp. was only abundant in the southern NB (Fig. 4b), while in the COI marker approach one species of *Paraeuchaeta* (*P. glacialis*) was among the dominant taxa throughout the NB and off the YP (Fig. 4e). Other copepods were rarely among the dominant taxa such as Heterorhabdidae, *Pseudocalanus* and *Oithona*. In the V4 and V9 datasets, undefined Calanoida contributed up to 24% of the NRSA.

Amphipoda was the most species-rich order among the Malacostraca with six species, of which the Hyperiidae with *Themisto abyssorum* and *Themisto libellula* were among the dominant taxa (Fig. 4). They were, however, not detected by V4 (S8). Next to these, we identified three abundant Euphausiacea species, which were abundant in the metabarcoding approaches (Fig. 4), and three species of Decapoda (Fig. 3a, S2). Other Arthropoda occurred only in low numbers (Fig. 3a, S2).

Cnidarians were the second taxon-richest phylum with eight species of five hydrozoan orders (Fig. 3a, S2), followed by the Echinodermata (Fig. 3a, S2)*.* Four Gastropoda (Mollusca) have been identified as the pteropods *Clione limacina* and *Limacina helicina* and one species each of the classes Nudibranchia and Neogastropoda. The Chaetognatha were represented by three species, while for the Chordata only one Appendicularia species (*Oikopleura* sp.) and one fish species (*Liparis fabricii*) could be identified. Only single taxa were found from the phyla Ctenophora, Annelida, Nemertea, Nematoda and Rotifera, respectively (Fig. 3a, S2).

At the BS station, the hydrozoan family Rhopalonematidae and the phylum Ctenophora (V4, V9), the hydrozoan genus *Euphysa* sp. (biovolume and biomass), Gastropoda (all approaches) and Actinopterygii (V9, COI; Fig. 4) were among the dominant taxa. Chaetognatha were among the dominant taxa in the NB and YP (V4, V9, COI; stations 70, 74, 78), i.e. the species *Eukrohnia hamata* (biovolume and biomass; stations 70, 71, 72, 73, 77, 78) and *Pseudosagitta maxima* (biovolume and biomass; stations 73, 76).

**Fig. 6:**
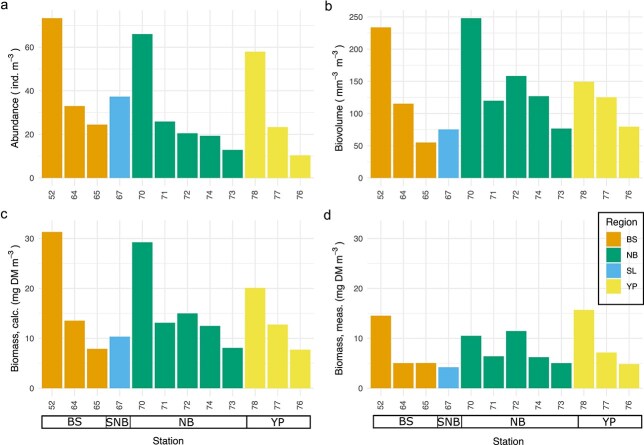
Total zooplankton at all stations, sorted by region and increasing latitude. (a) Abundance (ind m^−3^), (b) biovolume (mm^3^ m^−3^), (c) biomass calculated from biovolume (mg DM m^−3^) and (d) biomass, measured from bulk samples (mg DM m^−3^). The stations are ordered by region and increasing latitude in each region.

Total zooplankton abundance ranged from 13 ind m^−3^ at station 76, the northernmost station of YP, to 94 ind m^−3^ at the shallowest station 52 of BS (Fig. 6a). The abundances were exceptionally high at the southern stations of BS (52), NB (70) and YP (78). Total biovolume and biomass ranged from 50 mm^3^ m^−3^ (BS) to 248 mm^3^ m^−3^ (NB station 70) and from 8.5 mg DM m^−3^ (station 65, BS) to 31 mg DM m^−3^ (station 52, BS), respectively. Within each of the three regions BS, NB and YP, the total abundance, biovolume and biomass decreased northwards. Although the calculated and the measured bulk biomass revealed the same geographical pattern, the calculated biomasses were considerably higher than the measured ones (Fig. 6c, d). It is likely that either the processing of the samples yielded an error or that the presence of one or a few large organisms skewed the biomass data. Thus, these data must be treated with care.

### Impact of environmental drivers on the zooplankton community

During the study period, the sea surface temperatures varied from −1.76 to −1.58°C. At the BS and SNB stations, the temperatures increased continuously with depth with highest temperatures at 100 m (2–3.2°C, Fig. 5a). At all other stations, the temperature increased only below 70-m depth. At most stations, the salinity was stable down to 20–30 m (<34.3) and then increased gradually at the NB and YP stations to maxima ranging from 34.25 to 34.74 at 100-m depth or strongly at the BS and SNB stations reaching 34.8 to 34.92 at 100-m depth (Fig. 5b). Fluorescence indicated chlorophyll maxima between 25- and 39-m depth, below the cold surface layer at all stations (Fig. 5c, S4). The water masses differed among the stations. At the shallow BS stations, AAW contributed considerably to the water in the upper 100 m (42–47%), while at the NB and YP stations mostly colder PSW was present (S4).

**Fig. 7:**
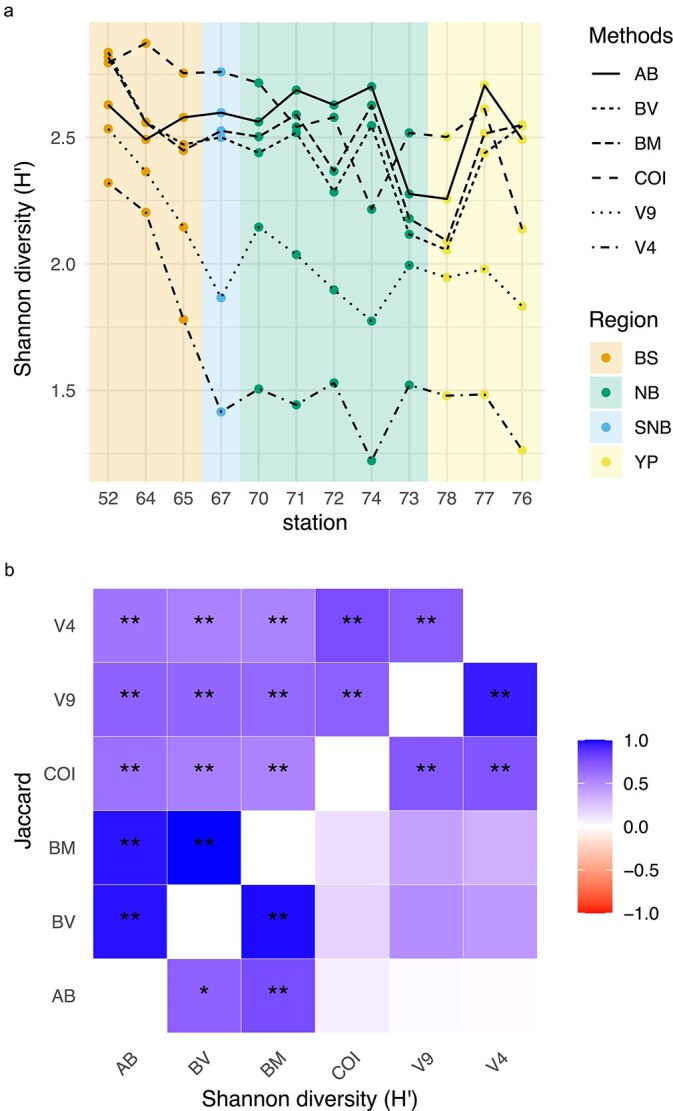
Diversity patterns in the study area: (a) Shannon diversity (H′) of image-based abundance (AB), biovolume (BV), biomass (BM) and normalized sequence read abundance of COI, 18S rRNA V9 and V4. The background is colored by regions (BS: Barents Sea, SNB: southern Nansen Basin, NB: Nansen Basin, YP: Yermak Plateau). Stations in each regions are sorted according to increasing latitude. (b) Correlation heatmaps of the pairwise Jaccard similarity indices (top) and the Shannon indices (bottom) among the different metabarcoding markers 18S rRNA V9, 18S rRNA V4 and COI with the image-based metrics abundance, biovolume and biomass. The correlations were computed using Pearson’s correlation coefficient. The colors in the heatmaps represent the strength of the correlation, with darker shades indicating stronger correlations. Asterisks show the significant correlations (^*^P < 0.05, ^**^P < 0.01).

A PCA of the environmental data revealed that temperature and salinity were correlated negatively with the first axis, while percentage share of PSW was positively correlated. The first axis also separated the BS and SNB stations from the NB and YP stations and explained already 65.9% of the variance in the environmental parameters (Fig. 5d). The second axis correlated with fluorescence and sea-ice thickness and explained another 17.7% of the variance. Shannon diversity indices were mostly correlated negatively with the first PCA axis (Fig. 5d). The Shannon diversity index for image-based abundance, however, showed the least correlation with the first axis and was closer to the second axis. A significant relationship among the Shannon indices between the metabarcoding and the imaging approaches could not be established (Fig. 7b), whereas the relationships among the presence/absence-based Jaccard diversity indices were significant (*P* < 0.01) (Fig. 7b, S10). It is worth noting that the Shannon indices for all approaches showed the same trend of decreasing diversity with increasing latitude (Fig. 7a).

**Fig. 8:**
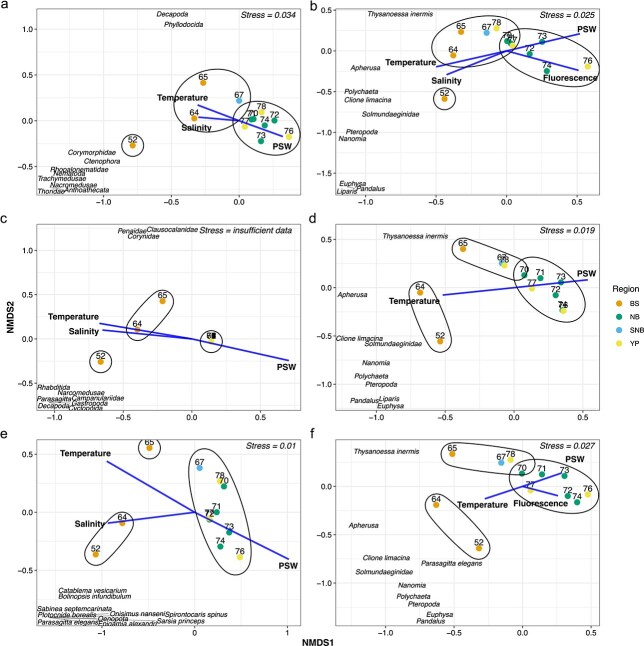
Non-metric multidimensional scaling (nMDS) for normalized sequence read abundance (NSRA) of (a) 18S V9, (c) 18S V4 and (e) COI, and for image-based relative (b) abundance, (d) biovolume and (f) biomass. Colors refer to the three regional station groups Barents Sea (BS), Nansen Basin (NB) and the Yermak Plateau (YP), and a single station in the southern Nansen Basin (SNB). Blue arrows show the significant environmental variables (*P* < 0.05) that influence the zooplankton community structure. Names show the 10 most influential taxa. Stations are grouped according to hierarchical clustering.

The spatial patterns were visualized with nMDS plots showing a similar distribution of the BS stations versus the NB and YP stations for each approach (Fig. 8). Generally, the two southern BS stations were separated from the other stations, while BS station 65 and SNB station 67 clustered in the image-based approaches together with the YP station 78. In the metabarcoding approaches, all three BS stations were separated from the other regions. In all nMDS plots, the stations of NB and YP overlapped greatly. For V4, the NSRA data from the NB, YP and SNB stations were almost identical as seen also in the taxonomic composition (Fig. 4).

Of the environmental variables, temperature and PSW were among the significant vectors in all approaches, being correlated negatively with the first nMDS axis, but not as close as in the PCA. Other significant environmental vectors were salinity in metabarcoding and image-based abundance. Fluorescence was a significant vector in the image-base abundance and biomass. As in the PCA, temperature and salinity were negatively correlated with the first axis and were pointing toward the BS stations, while PSW was positively correlated with the first axis and was important for the NB and YP stations. All significant environmental parameters were closer correlated with the first axis than with the second axis (Fig. 8). The absence of sea-ice thickness as an environmental vector indicates that this parameter had no significant impact on the zooplankton community during the present study. The nMDS plot also visualizes the 10 taxa that mostly explain the distribution of the communities. All taxa were on the negative side of the first axis, indicating a relationship with the BS stations. The taxa differed greatly among the six plots; however, at least 50% of these taxa belonged to gelatinous phyla (Cnidaria, Ctenophora, Mollusca, Polychaeta, Chaetognatha).

## DISCUSSION

In the present study, we investigated the diversity and the quantity of zooplankton communities in the epipelagic Arctic Ocean using a combination of multi-marker metabarcoding and image-based analysis to gain a deeper understanding of the community structure and spatial distribution of species and their ecological significance. All methods revealed similar patterns in zooplankton community structure and environmental drivers despite differences in taxonomic resolution and diversity. This suggests that the choice of markers and primers, sequencing depth and image resolution are sufficient to recover the epipelagic Arctic zooplankton community, specifically the community in the northern Barents Sea (BS) and the Nansen Basin (NB).

### Species identification and occurrence

The COI marker outperformed the 18S V4 and V9 barcodes as well as the image-based identification in terms of the number of identified taxa and the taxonomic resolution. COI was the only barcode that identified the community down to species level, while the 18S markers resolved taxa mainly to order level, and rarely to family or genus level (this study, Questel *et al.*, 2021). The image-based approach reached an intermediate taxonomic resolution. The successful species-level identification by COI compared to other markers and imaging tools has also been demonstrated in previous zooplankton metabarcoding studies (Clarke *et al.*, 2017; Djurhuus *et al.*, 2018; Stefanni *et al.*, 2018; Berry *et al.*, 2019; Carroll *et al.*, 2019; Ershova *et al.*, 2024; Novotny *et al.*, 2025).

The community was largely composed of epipelagic zooplankton with distribution ranges in the Central Arctic Ocean (based on a comparison to OBIS). We identified 29 out of 36 epipelagic species reported from the NB, plus 7 mesopelagic species (Kosobokova *et al.*, 2011). Among copepods, 15 out of the 18 species in our study corresponded to those listed by Kosobokova *et al.* (2011). Compared to earlier studies from the BS to the central NB (Hirche and Mumm, 1992; Mumm, 1993), we found overall more species (58 vs. 45). However, only 29 species overlapped with Mumm (1993), including 13 copepod species. Along the transect, we also recorded species known from neighboring regions such as Svalbard, particularly among the copepods (Weydmann *et al.*, 2014; Gluchowska *et al.*, 2016; Søreide *et al.*, 2022). These comparisons suggest that our study captured a broad representation of the epipelagic fauna expected in the Arctic Ocean. We note, however, that due to the larger mesh size (320 μm) and the applied 500-μm fractionation threshold our dataset mainly covers larger zooplankton. Consequently, we missed small-sized species such as *Oithona similis* and *Microcalanus* spp., which are known to greatly outnumber larger calanoids in the Arctic Ocean (Daase and Eiane, 2007; Gluchowska *et al.*, 2016; Cornils *et al.*, 2022).

The presence of the temperate copepod *Pseudocalanus mimus* (Frost, 1989), identified molecularly on the BS shelf, could not be confirmed by imaging due to the interspecific morphological differences in this genus (Frost, 1989). To our knowledge, *P. mimus* has not previously been reported from our study area (OBIS; Kosobokova *et al.*, 2011; Aarbakke *et al.*, 2011, 2014, 2017). A misassignment of sequence data is unlikely because (1) comprehensive COI reference data for regional *Pseudocalanus* species are available (Aarbakke *et al.*, 2011, 2014, 2017), and (2) our sequence matched those identified morphologically and by COI from the Eastern North Pacific and the Pacific Arctic (Questel *et al.*, 2016). The species’ distribution center in the Pacific Arctic, and its potential advection to the northern BS via the Siberian shelf or Beaufort Gyre/Transpolar Drift system, may explain its occurrence in our study area.

In contrast to copepods, only three and two species of Cnidaria in our study overlapped with Kosobokova *et al.* (2011) and Mumm (1993), respectively. This likely reflects the higher identification success of molecular methods compared to morphological ones, as shown in other Arctic studies (Murray *et al.*, 2024). For example, previously unidentifiable siphonophore fragments from images could be assigned to *Nanomia cara* through parallel metabarcoding. The *Nanomia* sequences from this study matched well with those of *N. cara* reported by Hosia *et al.* (2024). This recent integrative study revealed high diversity within the *Nanomia* genus and highlighted past misidentifications, which may also have affected sequence reference databases (Hosia *et al.*, 2024). The match therefore validates the identification of *N. cara* and confirms its presence in our study area consistent with its known occurrence in Atlantic water masses of the Arctic (Hosia *et al.*, 2024).

### Comparison of diversity between metabarcoding and imaging

Our data revealed that the Shannon diversity of the COI dataset was higher than that of the 18S markers (V4, V9), highlighting COI as the most suitable molecular marker to capture community diversity. Although COI recovered the highest species richness, its Shannon diversity was similar to that of the imaging approach, likely because the strong overrepresentation of *C. hyperboreus* in COI sequence data counteracted the higher richness. In comparison, the 18S markers showed considerably lower diversity.

The Jaccard diversity was significantly correlated among all approaches, reflecting consistent detection of dominant and key taxa across methods. However, Shannon diversity was only correlated among molecular approaches and among imaging-derived metrics. This is not surprising, as biovolume and biomass are calculated from the same image dataset. Interestingly, the Shannon indices from metabarcoding NSRA correlated more strongly with biovolume/biomass than with abundance, a pattern also observed in previous studies (Schroeder *et al.*, 2020; Ershova *et al.*, 2023). Such correlations, however, can vary across taxa (Novotny *et al.*, 2025) and thus may distort whole-community comparisons.

The COI-based community composition further illustrates this bias, as large species such as *C. hyperboreus* and *Paraeuchaeta glacialis* were overrepresented compared to imaging, while smaller-sized taxa such as *Pseudocalanus* spp. and *C. finmarchicus* were likely underestimated in sequence data.

### Community structure

In all approaches the zooplankton community was dominated by Calanidae, specifically *C. hyperboreus* and *C. finmarchicus*, confirming previous findings (Thibault *et al.*, 1998; Daase and Eiane, 2007; Kosobokova and Hirche, 2009; David *et al.*, 2015). In line with earlier studies (Hirche and Mumm, 1992), the relative abundance and biomass of *C. finmarchicus* decreased northwards while that of *C. hyperboreus* increased. Total zooplankton abundance and biomass also declined with latitude.

However, the relative shares of these species differed between methods: *C. finmarchicus* was more abundant in image analysis, whereas *C. hypberboreus* dominated in COI metabarcoding. This may reflect the correlation of metabarcoding with biomass (Ershova *et al.*, 2023) or taxon-specific amplification efficiency (PCR/primer bias) favoring *C. hyperboreus* (Coguiec *et al.*, 2021). Conversely, the imaging approach may have overestimated *C. finmarchicus* as morphological identification of *C. finmarchicus* and *C. glacialis* based on the prosome length is hampered by size overlaps (Choquet *et al.*, 2018). Nevertheless, *C. glacialis* was only abundant at the BS stations in both datasets, suggesting that prosome length–based identification does not strongly underestimate its presence in the NB.

Total biomass in our study aligns with previous Arctic estimates (Thibault *et al.*, 1998; Kosobokova and Hirche, 2009). Overall, species identification, taxonomic composition, abundance and biomass were consistent with earlier microscopic counts, highlighting the usefulness of combining metabarcoding and imaging for analyzing zooplankton community structure, with added value over morphology alone.

### Environmental drivers

Environmental variables are known to influence Arctic zooplankton communities (Gluchowska *et al.*, 2016), and our integrated approach confirms this. Regional differences, such as between the BS and NB, were well captured by both metabarcoding NSRA and imaging counts, revealing similar spatial patterns and environmental drivers. An earlier study also reported congruent drivers between metabarcoding and microscopy (Ershova *et al.*, 2024).

In our study, the BS epipelagic zooplankton community is directly influenced by modified Arctic Atlantic water (AAW), reaching up to 50-m depth, while the presence of both Polar Surface Water (PSW) and AAW in the upper 100-m depth likely drives spatial differences in taxonomic composition. nMDS analysis confirmed the significant effects of temperature and PSW contribution, consistent with earlier observations (Hirche and Mumm, 1992; Daase and Eiane, 2007; Hop *et al.*, 2021). Zooplankton biomass and biovolume were highest in the core of the Atlantic inflow and decreased with diminishing AAW influence, mirroring abundance patterns.

Surface salinity and Chl *a* concentration also contributed to latitudinal differences in species composition, in line with Van Engeland *et al.* (2023), although in that study these effects reflected temporal variability. Fluorescence as a proxy for Chl *a* had little effect on spatial patterns, with all stations showing chlorophyll maxima between 20 and 40 m, typical of late spring and summer in the Arctic after the surface bloom had declined (Ardyna *et al.*, 2013).

### Evaluation of the metabarcoding approaches

Previous studies on marine zooplankton metabarcoding (Clarke *et al.*, 2017; Djurhuus *et al.*, 2018; Stefanni *et al.*, 2018; Carroll *et al.*, 2019; Novotny *et al.*, 2025) recommend combining evolutionary independent markers in a multi-marker approach to improve species detection and reduce false negatives (Zhang *et al.*, 2018; Berry *et al.*, 2019, 2023). In our direct comparison of barcodes on dedicated zooplankton samples, however, COI alone performed very well: it covered most taxa, resolved species-level identification and captured the same spatial patterns as other markers. We did not observe broader taxonomic coverage with 18S rRNA V4 or V9 compared to COI, an argument often made for multi-marker approaches (Bucklin *et al.*, 2019; Blanco-Bercial, 2020; Questel *et al.*, 2021).

However, COI does also have limitations: it missed some rare groups (Rotifera, Nematoda) and Appendicularia, which are ecologically important in polar pelagic ecosystems, as shown by imaging and previous studies (Ehrlich *et al.*, 2020; Jaspers *et al.*, 2023; Schaafsma *et al.*, 2024). The 18S markers detected Appendicularia, albeit in very low numbers, but did not expand overall taxonomic coverage and missed other key taxa such as Amphipoda (V4). However, V4 does not generally fail to detect amphipods—they can be detected by selecting different primers (Zhan *et al.*, 2013; Zhang *et al.*, 2018; Ohnesorge *et al.*, 2023). Multi-marker approaches can mitigate primer bias and marker-specific failures, but in well-characterized regions, focusing on a single marker may be sufficient, when resources are limited.

The reliability of sequence-based diversity analysis depends on coverage in reference databases. In our study, taxonomic assignments using different algorithms and open-access databases (see S11) showed high agreement, indicating sufficient coverage for epipelagic Arctic zooplankton. Nevertheless, additional sequences for local species, such as *P. maxima*, from neighboring regions (Kulagin and Neretina, 2017), should be generated to improve assignment reliability (Questel *et al.*, 2021). We highly recommend expert plausibility checks of species-level assignments, especially for closely related taxa. For example, in the genus *Calanus*, some ASVs could not be assigned to species with the RDP classifier. Using local BLAST and NCBI GenBank, top hits included nine *C. glacialis* and one *Calanus marshallae* (AF332768; Hill *et al.*, 2001) both with valid identifications and known distributions (Hill *et al.*, 2001; Lizano *et al.*, 2022). While the genetic similarity between the two sibling species has been documented (Hill *et al.*, 2001; Ashjian *et al.*, 2017), recent *de novo* transcriptome sequencing supports their status as distinct species (Lizano *et al.*, 2022). This example highlights the importance of careful evaluation when assigning closely related species in metabarcoding studies. For nuclear markers, we also observed occasional misassignments of *Calanus* ASVs to *Neocalanus cristatus* when using the PR2 database, underscoring the need for cross-validation with multiple reference sources. Similarly, for the chaetognaths *E. hamata* and *Eukrohnia bathyantarctica*, low COI divergence led to ambiguous assignments; we assigned these ASVs to *E. hamata*, as *E. bathyantarctica* is not known from our area (Jennings *et al.*, 2010; Ershova *et al.*, 2023).

In summary, the MetaZooGene database with the RDP classifier is suitable for identifying epipelagic Arctic zooplankton. Cross-validation with alternative algorithms and non–marker-specific databases is recommended to resolve assignment ambiguities, especially in closely related species.

### Evaluation of the zooplankton image analysis

Overall, the imaging data provided more accurate quantification of individuals and the relative contribution of taxa to the community compared to the metabarcoding approaches. Sequence read abundances, however, can also reflect biomass, as shown for COI in this and previous studies (Ershova *et al.*, 2023; Novotny *et al.*, 2025).

Taxonomic resolution in imaging was lower than in COI metabarcoding. While common groups such as Calanoida and Chaetognatha were resolved well, rare taxa were less reliably identified, indicating that microscopic studies still provide higher resolution for less abundant species.

Nevertheless, image-based analysis offers several advantages over traditional microscopy: (1) automated extraction of object-level size and shape metrics for direct biomass estimation, (2) creation of a digital archive for long-term reference or re-analysis, and (3) extraction of life-history traits such as lipid storage, reproductive development and body size. Imaging also ensures consistent processing quality and reduces the need for extensive taxonomic training across multiple personnel. While manual validation of automated classifications remains necessary, previous studies have shown that the overall time investment remains lower than for traditional microscopy of comparable sample sizes (e.g. Plum *et al.*, 2021; Cornils *et al.*, 2022).

### The added value of a joint approach

The parallel application of metabarcoding and imaging enabled a more comprehensive characterization of zooplankton communities than either method alone. COI metabarcoding provides high-resolution species identification, while imaging allows for quantification. By combining both methods on split samples (as demonstrated here and in Matthews *et al.*, 2021), methodological biases can be reduced, and complementary insights into community composition and function can be gained.

In this study, both methods independently captured similar spatial patterns in community structure and identified the same key environmental drivers. Their integration, however, allowed for a more complete picture of Arctic zooplankton biogeography. Differences in the taxonomic resolution and species detection between methods (taxonomic strength) highlight methodological sensitivities and ecological nuances that become only apparent through cross-validation. Moreover, the combination of relative sequence data and organism-level size metrics supports not only presence–absence assessments, but also biomass and biovolume estimates—key components for ecosystem modeling (e.g. Schroeder *et al.*, 2020). The main advantages of each approach and of their integration are summarized in Table IV.

**Table IV:** Potentials and limitations of zooplankton metabarcoding and image analysis of zooplankton samples

**Aspect**	**Multi-marker metabarcoding**	**Image-based analysis**	**Combined approach**
Detected taxa	Higher taxa numbers on potentially all taxonomic levels	Lower taxa numbers; limitations especially at genus and species level	Broadest taxonomic coverage, including congeneric and morphologically ambiguous taxa. Chance for reverse taxonomy
Taxonomic resolution	Species-level identification possible (especially with COI); high resolution for small/early stages	Limited by image resolution and perspective; species level often not possible	Morphological and genetic comparison allows for adjustments and complementations
Quantification	Relative abundance based on read numbers, but not directly linked to individuals or biomass	Direct quantification of individuals, biovolume and biomass	Enables linkage of genetic diversity to quantitative metrics
Marker-specific detection	Different markers resolve on different taxonomic levels and identify/miss specific groups, e.g. COI for species level, 18S for broad detection	Not dependent on genetic markers, but limited by image quality and the preserved morphological condition	Can compensate for marker limitations (e.g. only 18S V4 identified Appendicularia but missed Amphipoda)
Taxon-specific strengths	Effective for fragmented or small organisms (e.g. Cnidaria, Ctenophora)	Effective for larger and distinct organisms (e.g. copepods, amphipods)	Improved detection across diverse taxonomic groups
Ecological insights	Enables molecular diversity indices (e.g. Shannon, Jaccard); strong biogeographic resolution	Captures patterns in abundance, biovolume and biomass	Supports robust ecological interpretations
Cost and effort	Higher laboratory and computational effort (DNA extraction, sequencing, bioinformatics)	Lower technical effort, but time-intensive manual identification	Resource efficient through complementary application
Limitations	DNA may come from detritus or partial organisms; no absolute abundance; primer bias misses some taxa	Limited detection of damaged or small organisms	Reduces method-specific biases and provides a more complete community profile

In conclusion, we recommend the combined use of metabarcoding and imaging wherever feasible. Their complementary strengths improve taxonomic resolution, quantification and trait-based interpretation, providing a robust framework for ecological studies and monitoring of plankton communities.

## Supplementary Material

Supplementary_data_revision_20250912_fbaf059(1)_rev

## Data Availability

All abundance, and biovolume, calculated dry mass and measured total dry mass data are available at PANGAEA (Cornils *et al.*, 2024), and the images can be viewed in the Ecotaxa project (https://ecotaxa.obs-vlfr.fr/prj/2534). Raw sequence data (fastq.gz files) and sample metadata were deposited in the European Nucleotide Archive (ENA) at EMBL-EBI under the accession number PRJEB82775 (https://www.ebi.ac.uk/ena/data/view/PRJEB82775).
