# Supplementary material for: Of sequences and images - diversity and quantity of Arctic epipelagic zooplankton by an integrative approach
Source: J Plankton Res. 2025 Nov 28;47(6):fbaf059. doi: 10.1093/plankt/fbaf059 (PMC12661942; doi:10.1093/plankt/fbaf059)
Supplement: Supplementary_data_revision_20250912_fbaf059(1)_rev [file supplementary_data_revision_20250912_fbaf059(1)_rev.docx]

# Supplementary Data

**S1:** Workflow on sample processing for Imaging analysis by Zooscan and multi-marker metabarcoding analysis by MiSeq sequencing.

**
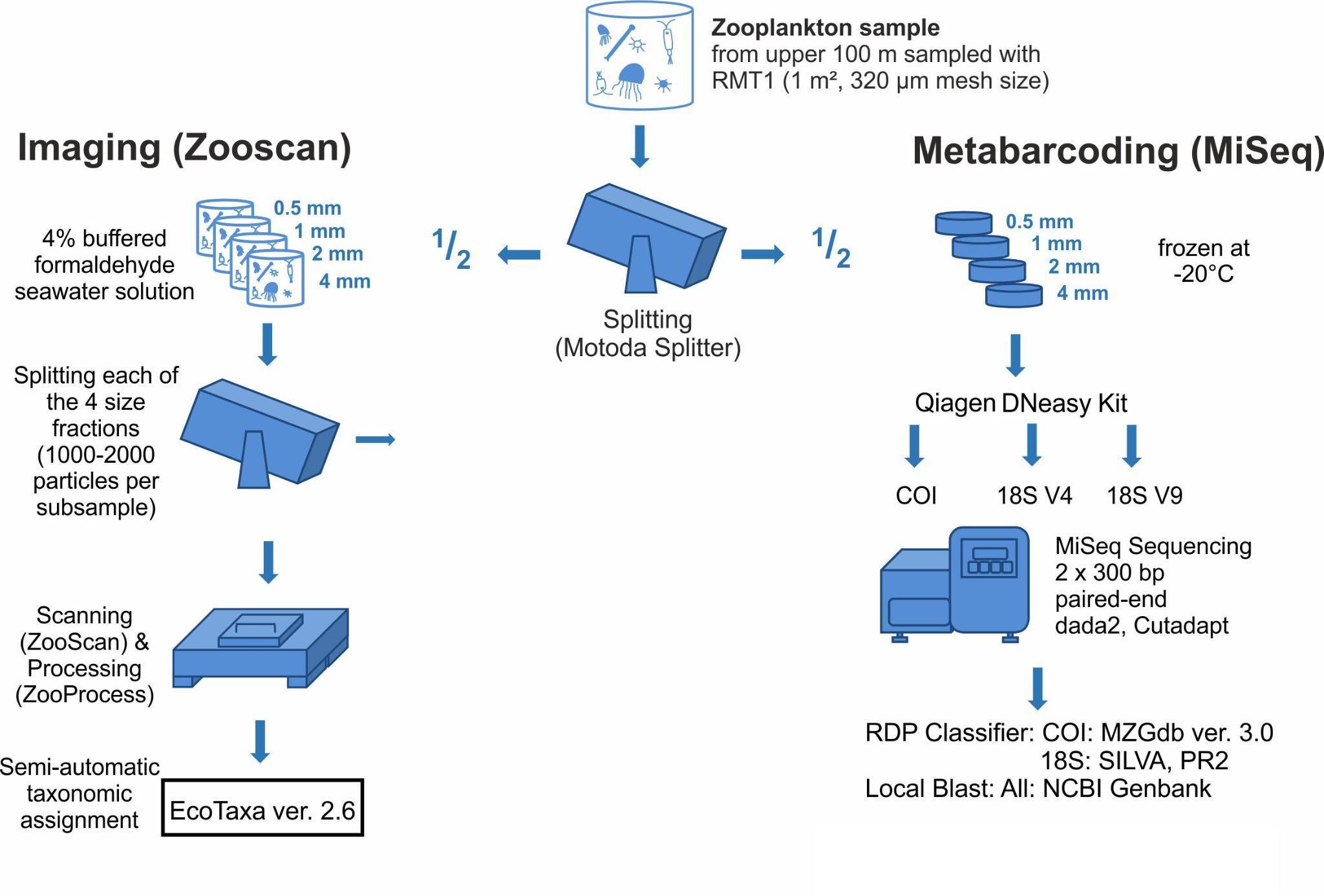
**

**S2:** Taxa of Arctic zooplankton identified by image analysis (*) and metabarcoding (+). The cross or the asterisk is always set at the taxonomic level with the highest taxonomic resolution.

| **Phylum** | **Class** | **Order** | **Family** | **Genus** | **Species** |
| --- | --- | --- | --- | --- | --- |
| Annelida | Polychaeta* | Phyllodocida | Syllida | *Epigamia* | *E. alexandri+* |
|  |  | Spionida+ |  |  |  |
| Arthropoda | Copepoda | Calanoida | Aetideidae | *Chiridius* | *C. obtusifrons*+* |
|  |  |  |  | *Jaschnovia* | *J. brevis+* |
|  |  |  | Calanidae | *Calanus* | *C. finmarchicus*+*  *C. glacialis*+*  *C. hyperboreus*+* |
|  |  |  | Clausocalanidae | *Pseudocalanus** | *P. acuspes+*  *P. mimus+*  *P. minutus+*  *P. moultoni+* |
|  |  |  | Euchaetidae | *Paraeuchaeta** | *P. glacialis+*  *P. norvegica* |
|  |  |  | Heterorhabdidae | *Heterorhabdus* | *H. norvegicus*+* |
|  |  |  | Metridinidae | *Metridia* | *M. longa*+*  *M. lucens+* |
|  |  |  | Scolecitrichidae | *Scolecithricella* | *S. minor*+* |
|  |  | Cyclopoida | Oithonidae | *Oithona** | *O. atlantica+*  *O. similis+* |
|  |  |  | Oncaeidae | *Triconia* | *T. borealis+* |
|  |  | Harpacticoida* | Tisbidae | *Tisbe* | *T. furcata+* |
|  | Malacostraca | Amphipoda | Hyperiidae | *Themisto* | *T. abyssorum*+*  *T. compressa+*  *T. libellula*+* |
|  |  |  |  | *Hyperoche* | *H. medusarum+* |
|  |  |  | Uristidae | *Onisimus* | *O. nanseni+* |
|  |  |  | Calliopiidae | *Apherusa* | *A. glacialis*+* |
|  |  | Decapoda | Thoridae | *Spirontocaris* | *S. spinus+* |
|  |  |  | Crangonidae | *Sabinea* | *S. septemcarinata+* |
|  |  |  | Pandalidae | *Pandalus** | *P. borealis+* |
|  |  |  | Penaidae+ |  |  |
|  |  | Euphausiacea | Euphausiidae* | *Thysanoessa* | *T. inermis*+*  *T. longicaudata*+*  *T. raschii+* |
|  |  | Isopoda*+ |  |  |  |
|  | Thecostraca | Scalpellomorpha | Lepadidae | *Dosima* | *D. fascicularis+* |
|  | Ostracoda* | Halocyprida | Halocyprididae | *Boroecia* | *B. maxima+* |
|  |  |  |  | *Discoconchoecia* | *D. elegans+* |
|  |  |  |  | *Metaconchoecia* | *M. skogsbergi+* |
| Chaetognatha | Sagittoidea | Phragmophora | Eukrohniidae | *Eukrohnia* | *E. hamata*+* |
|  |  | Aphragmophora | Sagittidae | *Parasagitta* | *P. elegans*+* |
|  |  |  |  | *Pseudosagitta* | *P. maxima*+* |
| Chordata | Actinopteri | Cottoidei | Liparidae | *Liparis** | *L. fabricii+* |
|  | Appendicularia | Copelata | Oikopleuridae | *Oikopleura*+* |  |
| Cnidaria | Hydrozoa+ | Anthoathecata | Corymorphidae | *Euphysa** | *E. flammea+* |
|  |  |  | Boreohydridae | *Plotocnide* | *P. borealis+* |
|  |  |  | Corynidae | *Sarsia* | *S. princeps+* |
|  |  |  | Pandeidae | *Catablema* | *C. vesicarium+* |
|  |  |  |  | *Halitholus* | *H. cirratus+* |
|  |  | Leptothecata | Campanulariidae+ |  |  |
|  |  | Narcomedusae | Solmundaeginidae | *Aeginopsis** | *A. laurentii+* |
|  |  | Trachymedusae | Rhopalonematidae | *Aglantha* | *A. digitale*+* |
|  |  | Siphonophorae* | Agalmatidae | *Nanomia** | *N. cara+* |
| Ctenophora | Tentaculata | Lobata | Bolinopsidae | *Bolinopsis* | *B. infundibulum+* |
|  |  | Cydippida+ |  |  |  |
| Mollusca | Gastropoda | Pteropoda | Clionidae | *Clione* | *C. limacina*+* |
|  |  |  | Limacinoidea | *Limacina* | *L. helicina+* |
|  |  | Nudibranchia | Onchidorididae | *Onchidoris* | *O. muricata+* |
|  |  | Neogastropoda | Mangeliidae | *Oenopota+* |  |
| Echinodermata | Asteroidea | Forcipulatida | Asteriidae | *Urasterias* | *U. lincki+* |
|  | Ophiuroidea | Euryalida+ |  |  |  |
|  |  | Ophiurida | Ophiuridae | *Ophiocten* | *O. gracilis+*  *O. sericeum+* |
|  |  |  |  | *Ophiura* | *O. sarsii+* |
|  |  |  | Amphilepidida | *Ophiopholis* | *O. aculeata+* |
| Nemertea | Palaeonemertea |  | Cephalotrichidae | *Cephalothrix* | *C. iwatai+* |
| Nematoda | Chromadorea | Rhabditida*+* |  |  |  |
| Rotifera | Eurotatoria | Ploima | Synchaetidae*+* |  |  |

**S3:** Arctic zooplankton identified from the imaging analysis from phylum to species level (from the inside out: phylum, class, order, family, genus, species).


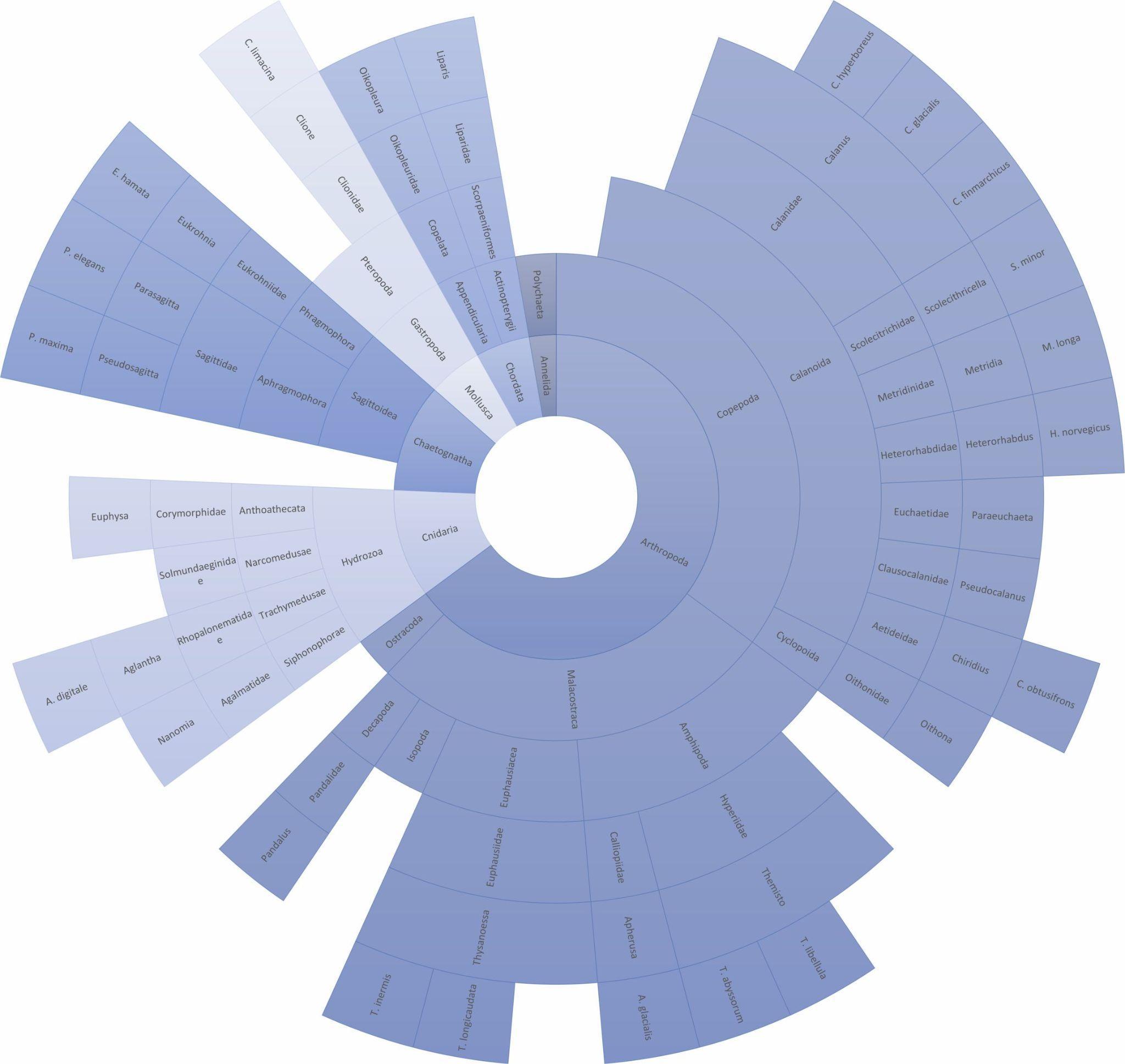


**S4:** Environmental variables of the zooplankton sampling stations. Temperature, Salinity and Fluorescence are averages from 0 to 100 m depth. DCM: Deep Chlorophyll Maximum (m), PSW%: share of Polar Surface Water, AAW%: share of Arctic Atlantic Water, AW%: share of Atlantic water.

| **Station** | **52** | **64** | **65** | **67** | **70** | **71** | **72** | **73** | **74** | **76** | **77** | **78** | **Comments** |
| --- | --- | --- | --- | --- | --- | --- | --- | --- | --- | --- | --- | --- | --- |
| Julian Day | 180 | 182 | 183 | 184 | 186 | 187 | 187 | 188 | 189 | 191 | 191 | 192 |  |
| Latitude | 80.826 | 81.414 | 81.595 | 81.954 | 83.119 | 83.334 | 83.501 | 83.714 | 83.468 | 82.490 | 82.245 | 82.050 |  |
| Longitude | 31.954 | 32.612 | 33.207 | 32.331 | 32.924 | 33.238 | 32.981 | 32.337 | 28.085 | 18.224 | 17.782 | 17.644 |  |
| Bottom depth (m) | 135 | 204 | 553 | 2818 | 3813 | 3903 | 3983 | 4022 | 4049 | 2278 | 2025 | 1849 |  |
| Region | BS | BS | BS | NB | NB | NB | NB | NB | NB | YP | YP | YP |  |
| CTD station | 50-2 | 65-1 | 65-1 | 67-3 | 69-4 | 71-1 | 73-1 | 73-1 | 74-1 | 76-1 | 76-1 | 78-1 | Heuze et al. (2018) |
| Temperature (°C) | 0.66 | -0.05 | -0.05 | 0.34 | -1.73 | -1.44 | -1.72 | -1.72 | -1.81 | -1.52 | -1.52 | -1.37 | mean calculated from Heuze et al. (2018) |
| Salinity | 34.55 | 34.45 | 34.45 | 34.42 | 34.25 | 34.38 | 34.38 | 34.38 | 34.36 | 34.23 | 34.23 | 34.20 | mean calculated from Heuze et al. (2018) |
| Fluorescence | 0.13 | 0.13 | 0.13 | 0.13 | 0.12 | 0.13 | 0.15 | 0.15 | 0.15 | 0.14 | 0.14 | 0.18 | mean calculated from Heuze et al. (2018) |
| DCM (m) | 24 | 37 | 37 | 32 | 37 | 48 | 39 | 39 | 38 | 22 | 22 | 22 | calculated from Heuze et al. (2018) |
| PSW (%) | 38 | 53 | 53 | 53 | 100 | 93 | 100 | 100 | 100 | 100 | 100 | 97 | derived from pot. T and Density from Heuze et al. (2018) |
| AAW (%) | 42 | 47 | 47 | 18 | - | 7 | - | - | - | - | - | 3 | derived from pot. T and Density from Heuze et al. (2018) |
| AW (%) | 21 | - | - | 29 | - | - | - | - | - | - | - | - | derived from pot. T and Density from Heuze et al. (2018) |
| Sea-ice thickness (m) | 1.19 | 0.97 | 2.29 | 1.29 | 1.97 | 2.44 | 1.01 | 2.56 | 1.77 | 1.94 | 2.44 | 2.04 | from Castellani et al. (2020) |

**S5:** Percentage of amplicon sequence variants (ASVs) (light grey) and sequence reads (dark grey) assigned to the different metazoan taxonomic ranks for A) 18S rRNA V4, B) 18S rRNA V9 and C) cytochrome c oxidase subunit I (COI).


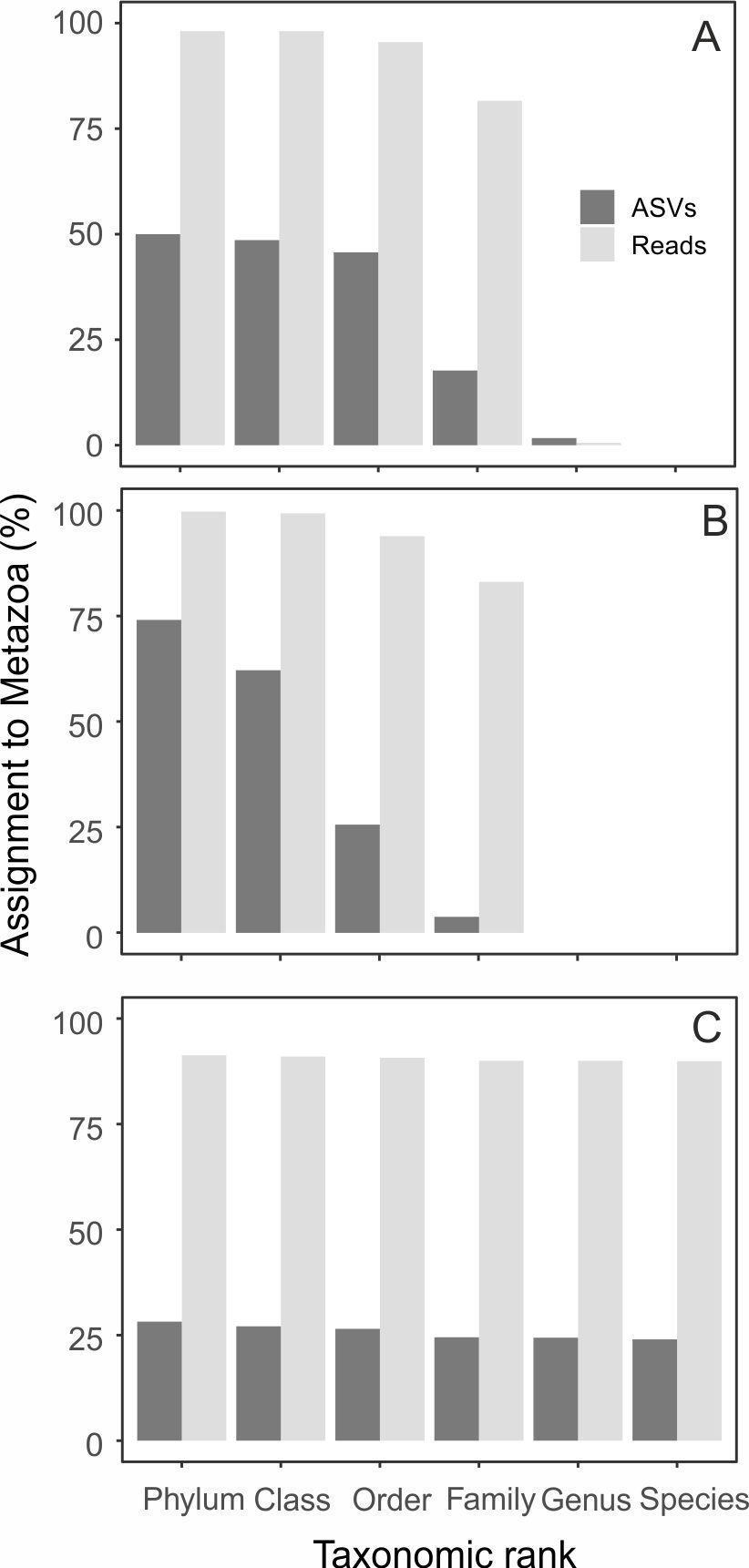


**S6:** Sequencing depth: ASV accumulation curves for the recovery of metazoan ASVs for A) 18S rRNA V4, B) 18S rRNA V9, and C) COI.


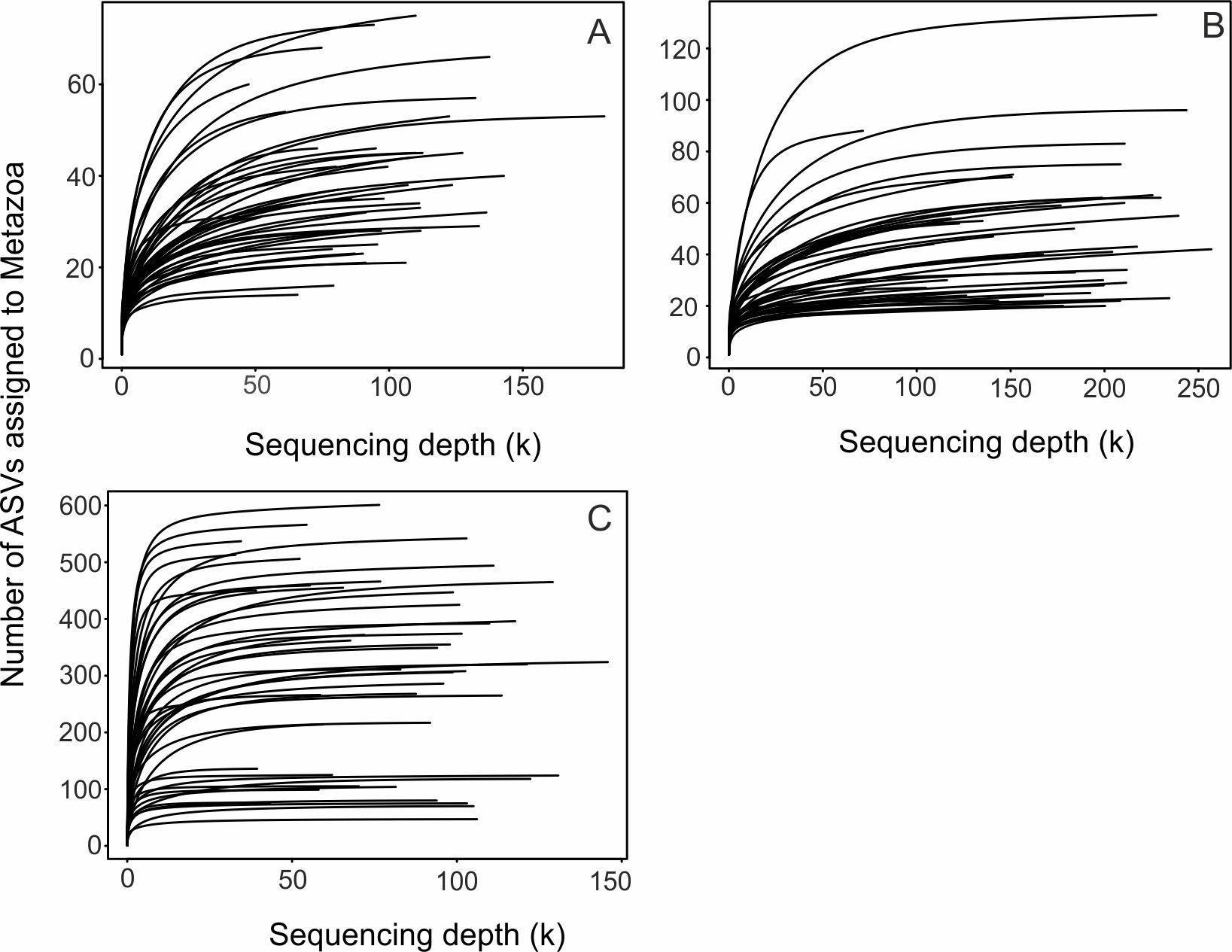


**S7:** Arctic zooplankton identified from the COI metabarcoding approach from phylum to species level (from the inside out: phylum, class, order, family, genus, species).


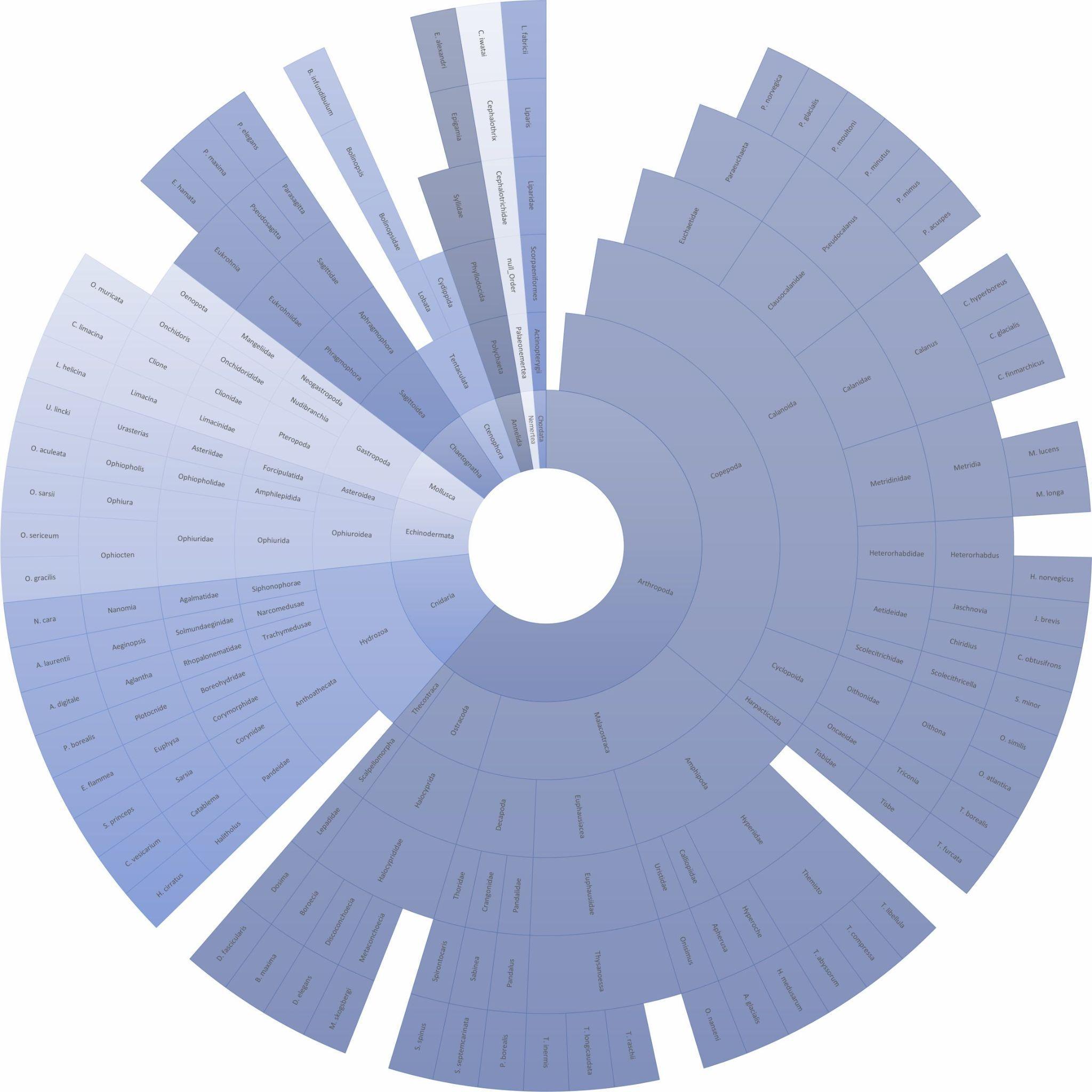


**S8:** Arctic zooplankton identified from the 18S rDNA V4 metabarcoding approach from phylum to genus level (from the inside out: phylum, class, order, family, genus).


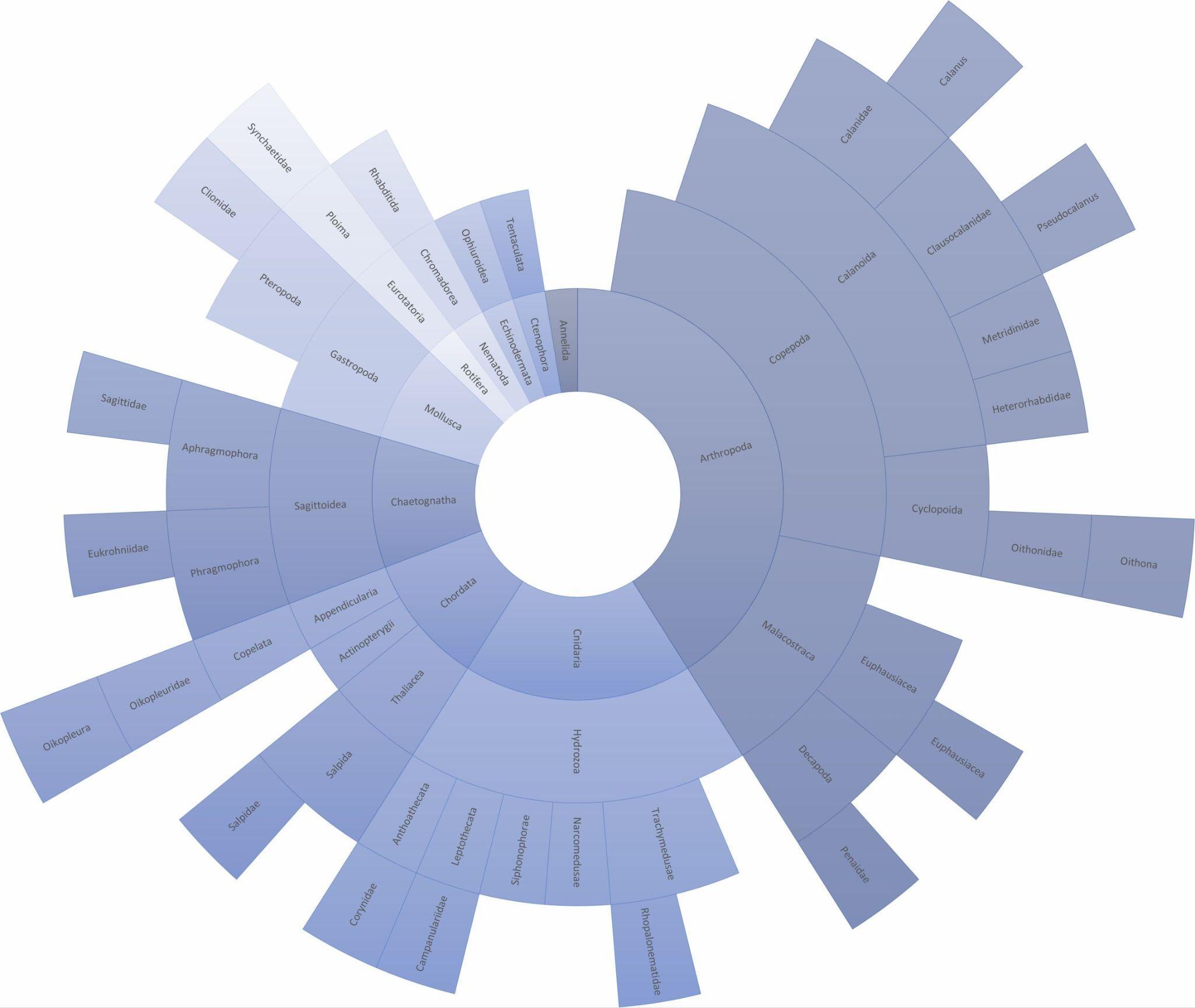


**S9:** Arctic zooplankton identified from the 18S rDNA V9 metabarcoding approach from phylum to family level (from the inside out: phylum, class, order, family).


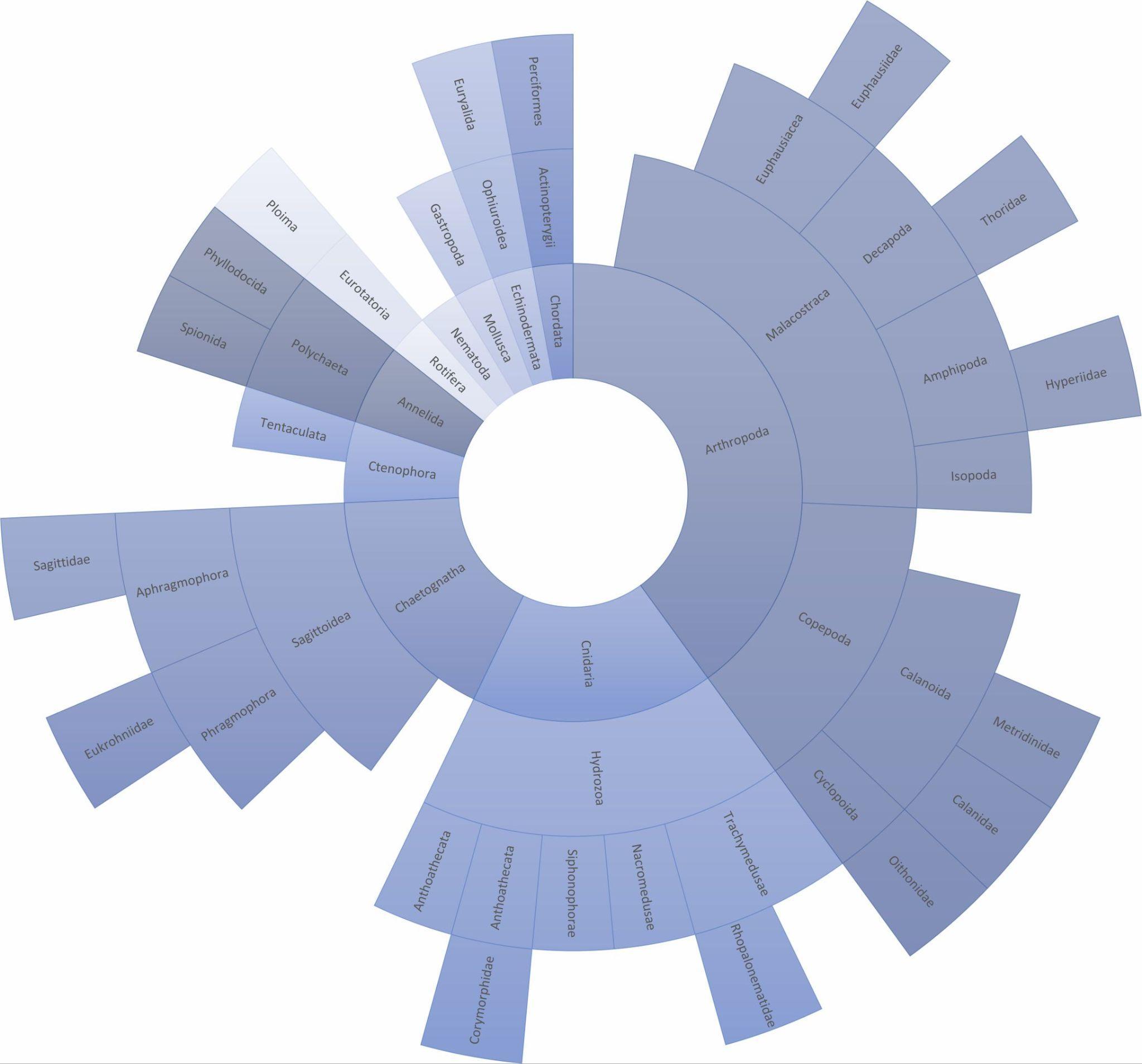


**S10:** Shannon diversity at the 12 stations for the three metabarcoding genes and the imaging parameters. Diversity was calculated for the lowest taxonomic level.

|  | 18S V4 | 18S V9 | COI | Abundance | Biovolume | Biomass |
| --- | --- | --- | --- | --- | --- | --- |
| 52 | 2.32 | 2.53 | 2.80 | 2.63 | 2.84 | 2.05 |
| 64 | 2.20 | 2.36 | 2.87 | 2.49 | 2.56 | 1.68 |
| 65 | 1.78 | 2.15 | 2.75 | 2.58 | 2.47 | 1.45 |
| 67 | 1.41 | 1.87 | 2.76 | 2.60 | 2.50 | 1.66 |
| 70 | 1.51 | 2.15 | 2.72 | 2.56 | 2.44 | 1.75 |
| 71 | 1.44 | 2.04 | 2.54 | 2.69 | 2.52 | 1.92 |
| 72 | 1.53 | 1.90 | 2.58 | 2.63 | 2.28 | 1.48 |
| 73 | 1.52 | 1.99 | 2.52 | 2.28 | 2.12 | 1.99 |
| 74 | 1.22 | 1.77 | 2.22 | 2.70 | 2.55 | 1.56 |
| 76 | 1.26 | 1.83 | 2.14 | 2.49 | 2.55 | 1.26 |
| 77 | 1.48 | 1.98 | 2.61 | 2.71 | 2.44 | 1.70 |
| 78 | 1.48 | 1.95 | 2.50 | 2.26 | 2.05 | 1.82 |

###

**S11:** Protocols for library preparation, sequencing, sequence processing and sequence data analysis including taxa assignment

Library preparation and sequencing

For the two nuclear markers, 18S rRNA regions V4 and V9, the mastermix had a final volume of 25 µL and contained 2.5 µL DNA template (5 ng/µL), 5 µL of each primer (1 ng/µL) and 12.5 µL of 2x Kapa HiFi HotStart Ready Mix (Kapa Biosystems, Roche, Germany). Thermoprofile was denaturation at 95°C (3 min), 25 cycles with 95°C (30 s), 55°C (30 s), 72°C (30 s) and final elongation at 72°C (5 min). Nuclear ribosomal 18S rRNA variable region V4 was amplified using the primer pairs 528iF and 964iR (Metfies et al., 2020) and V9 by using the primer pairs 1380F and 1510R (Amaral-Zettler et al., 2009). The PCR reactions for COI were prepared using the illustra PuRe-Taq Ready-To-Go PCR Beads (GE Healthcare), to which 2.5 µL DNA template (5 ng/µL), 21.5 µL water and 0.5 of each primer (20 pmol/µL) were added for a final reaction volume of 25 µL.

Amplification was conducted by using the degenerated primer pairs mlCOIintF-XT (Wangensteen et al., 2018) and jgHCO2198 (Geller et al., 2013) and a thermoprofile with a denaturation step at 95°C (15 min), 30 cycles with 94°C (30 s), 42°C (90 s), 72°C (60 s) and a final elongation at 72°C (10 min) (Ohnesorge et al., 2023). PCR triplicates per sample were pooled and were checked on a 1.5% agarose gel (GelRed Nucleic Acid Stain 10 000 x in water) and 1 kb DNA ladder (biotechrabbit ready to use). Sample purification was conducted using the AMPure XP beads (Beckman Coulter) according to the protocol by adding 20 µL beads to the PCR products and two washing steps with 80% ethanol. The indexing was performed with Nextera XT Index Kit v2 Set A according to the manufacturer’s protocol with a mastermix of 5 µL DNA template, 5 µL of each primer, 25 µL of 2 x KAPA HiFi HotStart Ready Mix and 10 µL of PCR grade water. The thermoprofile started with a denaturation step at 95°C (3 min) followed by eight cycles at 95°C (30 s), 55°C (30 s) and 72°C (30 s) and a final elongation at 72°C (5 min). The indexed PCR products were purified according to the protocol by adding 56 µL AMPure XP beads, two washing steps with 80% Ethanol and resuspended by using 27.5 µL 10 mM Tris pH 8 buffer.

For normalization and pooling of samples, DNA concentrations of all samples were determined using a fluorometer (QuantiFluorR dsDNA System, Promega, USA) including blanks. Samples were diluted to a final concentration of 4 nM with 10 mM Tris pH 8.5 buffer and pool. Per run, 96 samples (including 15% Phi) were sequenced on an Illumina MiSeqTM sequencer (Illumina, USA) with 2×300 bp paired-end reads at a target sequencing depth of 120.000 reads per sample.

Sequence Processing

Sequences from MiSeq sequencing run were demultiplexed and FASTQ sequence files generated by using the ‘Generate FASTQ’ workflow of the MiSeq sequencer software. Demultiplexed sequence data have been processed using the dada2 pipeline (Callahan et al., 2016) and cutadapt (ver 1.18, Martin, 2011) in R (ver. 4.1.1106, R Core Team, 2016). Read quality profiles of the raw and the processed files have been visualized and inspected.

We pre-filtered the sequence data for all three markers at a minimum length of 50 bp in the dada2 pipeline. We have also adjusted additional parameters for the 18S rRNA sequence data (maxN=0, truncQ=2, minQ=2). Primers are cut at 5´and 3´end with a minimum overlap of 20 bp (min_overlap=20) and a maximum error rate of 0.125 (max_error_rate_0.125), disallowing insertions and deletions entirely (--no-indels). The quality filtration of sequences was conducted with no allowances for N (maxN=0) and reads that match against the phiX genome are discarded (rm.phix = TRUE). For quality filtration of COI we truncated the sequences at 200 bp with maximum expected error of 2.0. For 18S rRNA V4 we used 220 bp for both forward and reverse and a maximum expected error of 2.1 and 2.1 (maxEE= 2.1, 2.1) and for V9 a truncate length of 130 bp and a maximum expected error of 1.3 (maxEE= 1.3,1.3). Sequences have been truncated allowing a Phred Score >30. For sample inference we chose the algorithm pool=”pseudo” to allow pseudo-pooling between individually processed samples. Sequences are merged with a minimum overlap of 20 bp (minOverlap=20). Potential chimeras were removed from the ASVs using the removeBimeraDenovo function (method: consensus).

Amplicon sequence variants (ASVs) of COI were assigned by using the in the dada2 pipeline implemented RDP Naive Bayesian Classifier (Wang et al., 2007) with a minimum bootstrap of 95 with the MetaZooGene database All Plankton Combo (All-World-Oceans) Mode-A, containing all data from the *All Zooplankton* and the *All Ichthyoplankton* combined files with data from all World Oceans (COI barcodes version 3.0, downloaded, 28.03.2022, Bucklin et al., 2021). The ASVs of 18S rRNA V4 and V9 were assigned using the RDP Naive Bayesian Classifier (Wang et al., 2007) and a minimum bootstrap of 100 each individually with Silva, PR2 and MetaZooGene database All Plankton Combo (All-World-Oceans) Mode-A, which contains all data from the *All Zooplankton* and the *All Ichthyoplankton* combined files with data from all World Oceans (18S rRNA barcodes, version 3.0, downloaded, 28.03.2022). All taxonomic assignments were cross-validated by using a local BLAST (ver. 2.13.0) and local GenBank nt database (download date: 03.02.2022) with screening the first Top 10 hits for COI and the first Top 30 hits for 18S rRNA data.

For COI missing assignments to species level with RDP Naive Bayesian Classifier (Wang et al., 2007) in combination with MZGdb have been supplemented by BLAST results when pairwise identity was >97% and query >300 bp query cover (e.g. Chaetognaths: *Eukrohnia hamata* (>98.4%, 313 bp), *Parasagitta elegans* (>99%, >300 bp); Copepods: *Calanus glacialis* (>97%, >300 bp as MZGdb only assigns to genus level). For the latter species, in some cases, RDP Naive Bayesian Classifier just identified down to genus level as best 10 hits comprising nine *C. glacialis* and one *C. marshallae* (AF332768 from Hill et al. 2001). Moreover, we renamed some species: *Metridia gerlachei* was renamed as NA as the ASV only matches with unpublished accession numbers from three sources (e.g. KC754399) and is assumed as misidentification in the sequence reference database (as *M. gerlachei* is an Antarctic species). The misassignment of *Ophiocten hastatum* was corrected by *Ophiocten gracilis* by the comparison to top 10 hits with Genbank.

18S V9 assignments with RDP Naive Bayesian Classifier in combination with MZGdb have been compared to SILVA and PR2 as well as to the best 30 hits from local BLAST in combination with NCBI GenBank. We added information on assignment when yielding high pairwise identify and query and when the first 30 hits showed uniform classification up to a certain taxonomic level. We added to family level (>99%, >130 bp query coverage) when the best 30 sequences match with the same species/genus (e.g. for the genera *Metridia* and *Calanus*). For the calanoid superfamily Clausocalannoidea no family was added as diverse families have been found among the best hits. Assigned species/genus level was deleted when more than one species have been assigned with high pairwise identity and high query coverage among the 30 best hits (e.g. in the assignment with PR2 all *Calanus* species are assigned as *Neocalanus cristatus*). At higher taxonomic levels, we added the order level when pairwise identity was >98% and phylum level when pairwise identity was >97%, both with query coverage of >130 bp.

For the 18S V4 fragment we added the genus level (at 100% pairwise identity, >300 bp query coverage) when all 30 best matches comprise the same genus. We have proceeded similarly with the other taxonomic levels with family (>98%, >200 bp), order and phylum (97%, <200 bp). Assignment to metazoan phylum level with MZGdb with matches on non-targets (e.g. dinoflagellates in PR2, SILVA and NCBI GenBank) have been renamed as NA.

Sequence data analysis

We deleted singletons (V4: 22, V9: 2, COI: in total 53 singletons across all samples) and checked that no rare species were deleted from the data set (all singletons are assigned to common species or were not assigned to a taxon (NAs)). Downstream analysis was conducted for assigned metazoan phyla only comprising Annelida, Arthropoda, Chaetognatha, Chordata, Cnidaria, Ctenophora, Echinodermata, Mollusca, Rotifera (only 18S rRNA), Nematoda (only 18S rRNA) and Nemertea (COI). We verified all species according to their distribution ranges (OBIS: obis.org) and based on literature research for their occurrence in the study area. Based on this analysis, all species identified in this study have their distribution range in the study area or in neighbouring areas.

References

Amaral-Zettler, L. A., McCliment, E. A., Ducklow, H. W. and Huse, S. M. (2009) A method for studying protistan diversity using massively parallel sequencing of V9 hypervariable regions of small-subunit ribosomal RNA genes. *PLoS ONE,* **4**, e6372.

Bucklin, A., Peijnenburg, K. T. C. A., Kosobokova, K. N., O'Brien, T. D., Blanco-Bercial, L., Cornils, A., Falkenhaug, T., Hopcroft, R. R. et al.. (2021). Toward a global reference database of COI barcodes for marine zooplankton. Marine Biology, 168, 78. https://doi.org/10.1007/s00227-021-03887-y

Callahan, B., McMurdie, P., Rosen, M. J., Han, A. W., Johnson, A. J. A. and Holmes, S. P. (2016) DADA2: High-resolution sample inference from Illumina amplicon data. Nature Methods, 13, 581–583. https://doi.org/10.1038/nmeth.3869

Geller, J., Meyer, C., Parker M. and Hawk, H. (2013) Redesign of PCR primers for mitochondrial cytochrome c oxidase subunit I for marine invertebrates and application in all-taxa biotic surveys. *Molecular Ecology Resources,* **13**, 851-861.

Martin M. (2011) Cutadapt removes adapter sequences from high-throughput sequencing reads. *EMBnet. journal*, **17**, 10-12. https://doi.org/10.14806/ej.17.1.200

Metfies, K., Hessel, J., Klenk, R., Petersen, W., Wiltshire, K.H. and Kraberg, A. (2020) Uncovering the intricacies of microbial community dynamics at Helgoland Roads at the end of a spring bloom using automated sampling and 18S meta-barcoding. *PLoS ONE*, **15**, e0233921. https://doi.org/10.1371/journal.pone.0233921

Ohnesorge, A., John, U., Taudien, S., Neuhaus, S., Kuczynski, L. and Laakmann, S. (2023) Capturing drifting species and molecules - Lessons learned from integrated approaches to assess marine metazoan diversity in highly dynamic waters. *Environ. DNA*, **5**, 1541-1556.

Wang, Q., Garrity, G. M., Tiedje, J. M. and Cole, J.R. (2007). Naive Bayesian classifier for rapid assignment of rRNA sequences into the new bacterial taxonomy. *Applied and Environmental Microbiology*, **73**, 5261–5267. https://doi.org/10.1128/AEM.00062-07.

Wangensteen, O. S., Palacin, C., Guardiola, M. and Turon, X. (2018) DNA metabarcoding of littoral hard-bottom communities: high diversity and database gaps revealed by two molecular markers. *PeerJ*, **6**, e4705.
